# A bead-based western for high-throughput cellular signal transduction analyses

**DOI:** 10.1038/ncomms12852

**Published:** 2016-09-23

**Authors:** Fridolin Treindl, Benjamin Ruprecht, Yvonne Beiter, Silke Schultz, Anette Döttinger, Annette Staebler, Thomas O. Joos, Simon Kling, Oliver Poetz, Tanja Fehm, Hans Neubauer, Bernhard Kuster, Markus F. Templin

**Affiliations:** 1 NMI Natural and Medical Sciences Institute at the University of Tübingen, 72770 Reutlingen, Germany; 2 Pharmaceutical Biotechnology, Eberhard-Karls-Universität Tübingen, Tübingen, 72770 Reutlingen, Germany; 3 Chair for Proteomics and Bioanalytics, Center of Life and Food Sciences Weihenstephan, Technische Universität München, 85354 Freising, Germany; 4 Center for Integrated Protein Science Munich, 85354 Freising, Germany; 5 Department of Obstetrics and Gynecology, Medical Faculty and University Hospital of the Heinrich-Heine University Duesseldorf, 40225 Düsseldorf, Germany; 6 Department of Pathology, University Hospital Tübingen, 72076 Tübingen, Germany; 7 Bavarian Biomolecular Mass Spectrometry Center (BayBioMS), Technische Universität München, 85354 Freising, Germany

## Abstract

Dissecting cellular signalling requires the analysis of large number of proteins. The DigiWest approach we describe here transfers the western blot to a bead-based microarray platform. By combining gel-based protein separation with immobilization on microspheres, hundreds of replicas of the initial blot are created, thus enabling the comprehensive analysis of limited material, such as cells collected by laser capture microdissection, and extending traditional western blotting to reach proteomic scales. The combination of molecular weight resolution, sensitivity and signal linearity on an automated platform enables the rapid quantification of hundreds of specific proteins and protein modifications in complex samples. This high-throughput western blot approach allowed us to identify and characterize alterations in cellular signal transduction that occur during the development of resistance to the kinase inhibitor Lapatinib, revealing major changes in the activation state of Ephrin-mediated signalling and a central role for p53-controlled processes.

## Abstract (web summary)

Dissecting cellular signalling requires the analysis of large numbers of proteins. Here the authors describe DigiWest, a high-throughput protein detection method that combines the concept of western and widely-used bead array systems that allows rapid quantification of hundreds of specific proteins.

## Graphical abstract

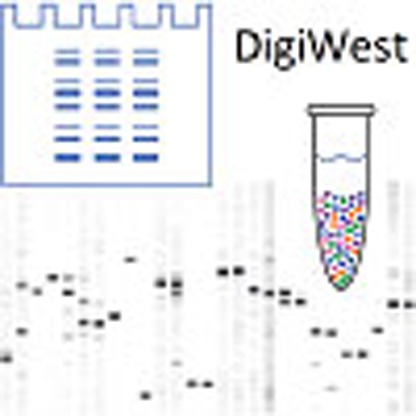

Significant progress has been made in understanding cellular activity through the molecular analysis of signalling pathways^1^. Pathway dysregulation and aberrant cellular signalling have been linked to diseases such as cancer^2^ and inflammatory disorders^3^. Although these breakthrough observations usually come from hypothesis-driven approaches, the development of new technologies^4^ and advances in automation now result in new possibilities for understanding cellular signalling^5^. By allowing the unbiased analysis of samples, mass spectrometry has had a massive impact on signalling research^6^, as have immunoassay-based approaches^7^. The proteome-wide generation of antibodies, driven by the Human Protein Atlas project^8^ and other groups^9^, opens up new possibilities and calls for immunoassay systems that are capable of performing multiple assays in parallel. Lower-throughput immunohistochemistry^10^, high-content screening technologies^11^ and reverse-phase protein microarrays (RPPAs)^12^ allow for ‘systems biology approaches' directly at the protein level. Information on the presence of a wide variety of proteins can be obtained and, by detecting post-translational modifications, the activation states of regulatory cascades can be interrogated. This type of information helps provide a better understanding of cellular processes. High-throughput technologies often come with compromises in data quality, and highly reliable approaches such as western blotting are often required to subsequently confirm the results.

Here, we describe an approach that enables a highly parallel analysis of protein expression and modification status by adapting the classical western blot^13^ to a bead-based microarray platform. Accurate size information is obtained using western blot, and the advantages of a bead-based microarray platform—high throughput and low material consumption—are utilized to increase the output of this classical method. The digital nature of the resulting data prompted us to name this approach ‘DigiWest'. We show that the sensitivity and reproducibility of this approach are as good as high-end western blotting systems and that the method is capable of providing high-resolution data on protein phosphorylation and expression. An analysis of the expression of almost 200 proteins in tumour cells collected by laser capture microdissection from primary human mammary carcinoma demonstrates the capabilities of this approach for characterizing limited sample material.

## Results

### Description of the DigiWest bead-based western blot

**Figure 1:**
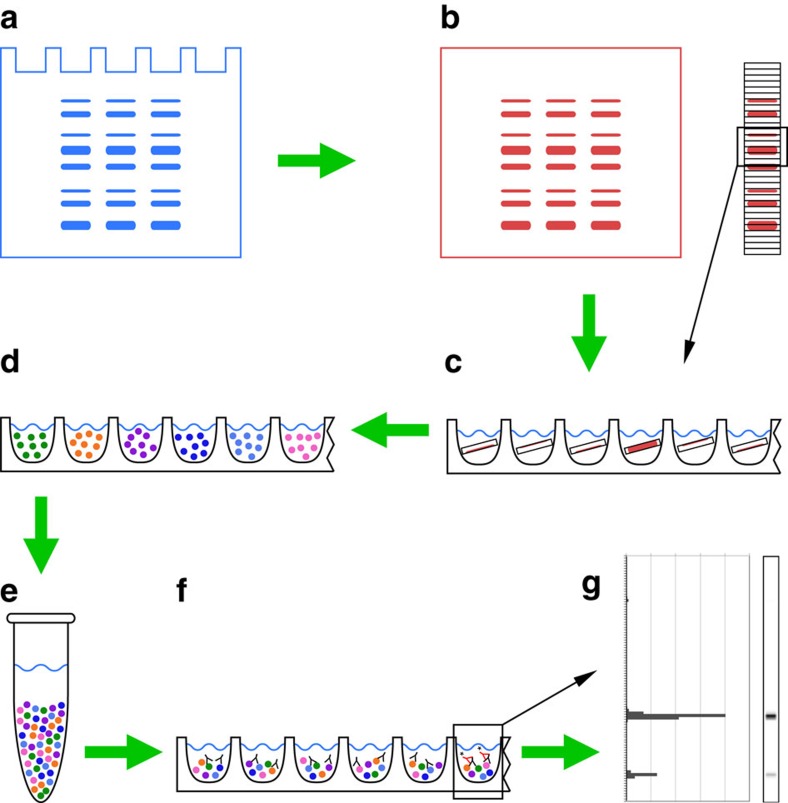
Bead-based western blot (DigiWest) workflow. (**a**) Protein separation by gel electrophoresis, usually SDS-PAGE. (**b**) Blotting of proteins to membrane and biotinylation of immobilized proteins directly on the membrane. (**c**) The cutting of sample lanes into 96 stripes to generate 96 molecular weight fractions immobilized on the membrane; elution of the proteins in 96-well plates. (**d**) Loading of biotinylated proteins onto 96 distinct Neutravidin-coated magnetic Luminex bead-sets. (**e**) Pooling into bead pools and reconstitution of the initial sample lane. (**f**) Immunoassay: aliquots of the generated bead pool (<0.5%) are incubated with western blot antibodies overnight before PE-labelled secondary antibodies are added for signal generation. (**g**) Readout using a Luminex instrument, reconstitution of the initial lane and data analysis.

The DigiWest approach combines standardized protein separation and western blotting with a multiplexed, bead-based immunoassay platform, such as the Luminex FlexMAP 3D system. As in standard western blotting, proteins are size separated via SDS-polyacrylamide gel electrophoresis (SDS-PAGE) and transferred to a blotting membrane (Fig. 1a,b). On this supporting membrane, all separated proteins are biotinylated before one sample lane is cut horizontally into 0.5-mm wide strips (Fig. 1c). Each strip carries immobilized proteins within a defined molecular weight range, and 96 protein fractions cover the range from 12 kDa to ∼400 kDa using a standard SDS-PAGE gel. The resulting strips are placed in individual wells of a 96-well plate and, through the addition of a harsh elution buffer, bound proteins are solubilized (Fig. 1c). After dilution of the eluate, Neutravidin-coated Luminex beads are added to each well, and the biotinylated proteins are immobilized on the bead surfaces. Because several hundred different colour-coded Luminex bead-sets are available, it is possible to add one distinct bead set to each of the 96 wells, resulting in a collection of distinguishable protein-loaded bead-sets from one sample (Fig. 1d). Thus, information regarding the molecular weight of the immobilized proteins is directly assigned to a defined colour code, and the mixing of the bead collection results in a bead pool that is equivalent to a reconstituted and digitized western blot lane (Fig. 1e). Sufficient beads for hundreds of antibody incubations can be routinely generated from 5–20 μg of protein, the amount commonly used for standard western blot analysis.

A small aliquot of the bead-set is used in a direct immunoassay, using one western blot antibody as a detection reagent followed by a Phycoerythrin-labelled secondary antibody for signal generation (Fig. 1f), and the sample is read on a flow cytometer (Luminex FlexMAP 3D). The discrete signals that are obtained for the different 96 bead populations represent the initial sample lane, each corresponding to one protein size fraction. After subtraction of background signals are plotted versus fraction number and a chromatogram like representation of the DigiWest data is obtained, in which protein bands are visualized as peaks (Fig. 1g).

**Figure 2:**
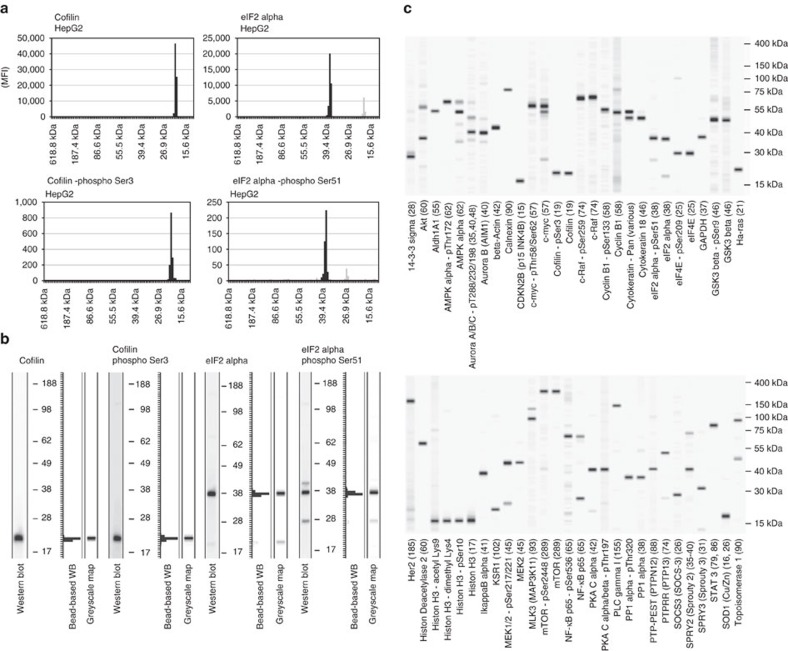
Comparison with western blot. (**a**) DigiWest: data obtained from 4 antibodies incubated with an aliquot of a bead pool loaded with HepG2 lysate. The 96 molecular weight fractions are represented as graphs. The specific signal is coloured dark grey. (**b**) The same antibodies used against the HepG2 lysate. Classical western blot (left), DigiWest data, illustrated as a graph (middle, same data as in **a**), and greyscale maps generated from DigiWest data (right, same data as in **a**). (**c**) Greyscale maps generated from 56 antibody incubations on the HepG2-lysate-loaded bead pool. A more comprehensive comparison of >100 antibodies on the western blot and DigiWest is provided in Supplementary Fig. 1.

In a first step of data evaluation the molecular weight distribution on the different bead populations is determined. Aliquots of the bead-set are probed with antibodies recognizing proteins with known molecular weight (for example, Histone H4-acLys8 11 kDa, Histone H3-acLys9 17 kDa, Bcl2 26 kDa, PP1 alpha 38 kDa, PKA C alpha 42 kDa, PPAR alpha 52 kDa, Akt 60 kDa, PLC gamma I 155 kDa and mTOR 289 kDa) and thereby specific signal in defined protein fractions is generated and assigned to a molecular weight. In analogy to SDS-PAGE these signals are used as molecular weight markers and by interpolation over the complete separation distance a molecular weight range can be assigned to each of the bead populations. No external molecular weight markers are required to determine the apparent weights of the detected proteins, the use of intrinsic markers ensures an excellent comparability between samples run on different gels. The discrete signals obtained for a given antibody on the 96 beads are now visualized as bar graphs in which fluorescence intensity is plotted against molecular weight (Fig. 2a); the graphs illustrate antibody-specific peaks at defined molecular weights correspond to the bands observed in a western blot. For the calculation of peak intensity contiguous signals at the correct size are identified and the integral is formed by totalling the fluorescence intensities while the local background is deducted. In contrast to western blotting, image processing is not required and since signal is generated using a calibrated flow cytometer a defined signal intensity with good reproducibility is obtained. In addition, the data can be transformed into an easily accessible graphical format. Converting the obtained signal intensities to a greyscale image and smoothing the image using a Gaussian filter results in a western blot-like data representation (Figs 1g and 2b). A visual examination of hundreds of such virtual western blots with dozens of samples lanes is possible and makes a fast way of identifying irregularities in the samples, whereas the calculated peak intensities present the numerical output for all proteins detected during one experiment. They allow the identification and description of differences between samples in a quantitative or semi-quantitative manner.

Since 500 colour-coded bead-sets are available, assays with a high degree of multiplexing are possible; 5 samples, each represented by 96 molecular weight fractions can be analysed simultaneously by generating one bead pool resulting in a substantial increase in throughput. Because DigiWest utilizes magnetic microspheres, it can be easily adapted to existing automation solutions, and dozens of bead pools can be handled in parallel.

### Characteristics of DigiWest

To compare the performance of DigiWest with that of classical western blotting, we used >100 thoroughly categorized western blot antibodies in the bead-based system. All selected antibodies were categorized and scored as described^14^ (see Supplementary Data 1) and most were found to be working in western blotting. The collection of antibodies used for the method comparison included (i) antibodies that detect proteins over the entire molecular weight range of the used gel system (ii) single-band antibodies and antibodies recognizing multiple bands, (iii) antibodies that result in high and low signals, and (iv) antibodies recognizing soluble and membrane-bound proteins, to test the capabilities of the system to deal with different types of antibodies. A bead pool was generated from 20 μg HepG2 lysate, sufficient to test all antibodies, and standard western blots were performed in parallel. The signals obtained from the DigiWest bead-sets were visualized as bar graphs in the shown examples; the Cofilin- and eIF2 alpha-specific antibodies, as well as antibodies recognizing their corresponding phosphorylated variants (Cofilin phospho Ser3; eIF2 alpha phospho Ser51; Fig. 2a), displayed distinct peaks at the expected molecular weights. Examining the western blot images and the greyscale maps generated from the DigiWest data enabled the direct comparison of the two systems (Fig. 2b; the complete set of pictures for the 104 tested antibodies is provided in Supplementary Fig. 1); a graphical overview of the output from 56 antibody incubations is presented in Fig. 2c. In addition, we could show that the biotin present in the immobilized proteins has no detectable influence on antibody binding (Supplementary Fig. 2). Differences in detected protein bands were observed for some antibodies; 21 of the 104 compared antibodies (20.2%) show visible differences between western blots and DigiWest. The observed discrepancies are mainly seen in the intensity of detected sidebands (differences observed for 13 antibodies corresponding to 12.5% of all antibodies) and differences in general background (3 antibodies corresponding to 2.9% of all comparisons). Differences in the generated band patterns were observed for 5 antibodies (4.8%), where high differences in signal intensity of the specific band were visible. Interestingly, they were found for binders that gave low signal intensities in both assay systems; unspecific binding and background signal become obvious under these conditions. Since slightly different assay and blocking buffers were used, these differences partially reflect the properties of the used blocking reagents (see the ‘Methods' section). A correlation analysis of western blot band intensities determined by a sensitive near-infrared fluorescence scanner (Li-COR Odyssey, Li-COR Instruments) with the DigiWest readout performed in identical buffers confirmed the visual perception, a Pearson's correlation coefficient *r* of 0.86 was calculated (see Supplementary Fig. 3). The direct comparison not only demonstrates the similarity of the two methods but also highlights obvious advantages of the newly established system. (i) Material consumption: 20 μg of sample, the amount usually used for a western blot, was used for generating DigiWest beads sufficient for 200 antibody analyses. (ii) Compatibility: all antibodies used for western blotting produce comparable results in the bead-based system. (iii) Size resolution: the exact determination of apparent molecular weights is possible. The resolution of the digitized data obtained by the DigiWest approach is directly comparable to the gel-based separation, the chosen sampling rate of 96 fractions has been optimized to directly compare conventional western blots and DigiWest data. Routinely, 8 or 16 samples are processed in parallel allowing the generation of the equivalent of several thousand western blot lanes in parallel and thereby bringing this classical approach to a significantly higher throughput.

### Performance and output of DigiWest

**Figure 3:**
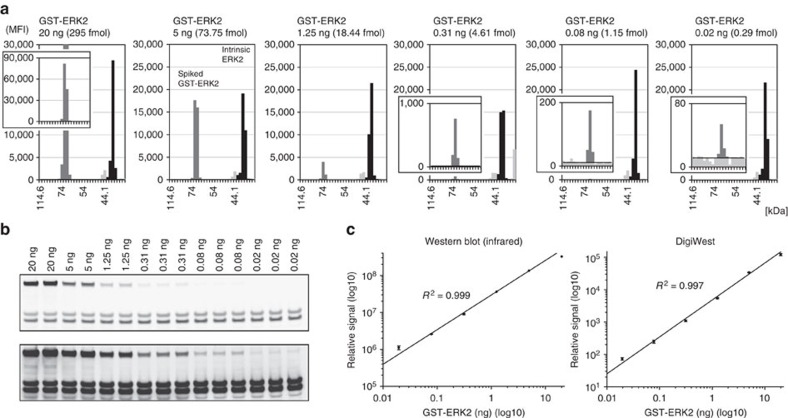
Reproducibility, sensitivity and signal linearity. A total of 10 μg of HepG2 lysate per lane was spiked with a 1:4 dilution series of GST-ERK2, from 20 ng to 20 pg. (**a**) Three replicates for each spike amount were used to generate DigiWest bead pools. An ERK1/2-specific antibody was used. One replicate for each spike amount is represented as graphs (all 18 lanes containing 3 replicates for each spike amount and 4 repeated measurements are provided in Supplementary Fig. 2). Intrinsic ERK2 is shown in dark grey, spiked GST-ERK2 in medium grey and intrinsic ERK1 in light grey. (**b**) The same samples on a western blot detected on a Li-COR Odyssey using the same primary antibody. The image is shown at two illuminations levels to depict the full GST-ERK2 dilution series. The three highest concentrations are only shown in duplicate because of the limited number of lanes on the gel. (**c**) Relative signal obtained from western blot (no error bars for the three highest concentrations) and DigiWest. Both axes are log10 scaled.

To demonstrate the sensitivity and dynamic range of the DigiWest approach and compare it with a sensitive western blot readout system, a spike-in experiment was designed. Different amounts of purified recombinant GST-ERK2 fusion protein (20 pg to 20 ng; 1:4 dilutions; prepared in triplicates) were spiked into 10- μg samples of HepG2 cell lysate. These mixtures were subjected to the DigiWest procedure, and aliquots of the resulting beads (equivalent to 100 ng total protein) were probed with a monoclonal anti-ERK1/2 antibody. The recombinant ERK fusion protein was detected as a discrete peak at a molecular weight of 68 kDa, whereas intrinsic ERK1 and ERK2 were observed at 44 and 42 kDa, respectively (Fig. 3a). Signal integration displayed good linearity, covering three orders of magnitude for the bead-based assay system. The observed signal linearity is in accordance with the high maximum-protein-loading capacity of the of used Neutravidin beads (Supplementary Fig. 4) that helps to avoid saturation and competition effects (Supplementary Fig. 5). Detection of the lowest used spike-in (20 pg GST-ERK2, 0.3 fmol) against a background of 10 μg HepG2 lysate was possible with a CV below 10% (see Supplementary Fig. 6 for the full data set).

To benchmark the results, classical western blots were performed using the identical sample materials, and a sensitive near-infrared fluorescence scanner (Li-COR Odyssey, Li-COR Instruments) was used for detection (Fig. 3b); both systems showed comparable characteristics and were capable of detecting 20 pg GST-ERK2 protein in the complex protein mixture. Using the spiked fusion protein as an internal standard, we were able to perform an absolute quantification of intrinsic ERK2. By using the signal intensities from all six spike concentrations, the amount of cellular ERK2 was found to be 7.1 fmol μg^−1^ (CV 9.6%) for the DigiWest approach and 6.23 fmol μg^−1^ (CV 6.4%) for the infrared western blot scan. These results agree well with published values^15^. Therefore, DigiWest exhibits comparable sensitivity and linearity with a high-end western blot system while requiring only 1/100th of the initial sample for one antibody incubation.

The experimental setup also provided information on the reproducibility of the method; 18 sample lanes (HepG2 lysate with 6 different amounts of spiked GST-ERK2, 3 replicates each) were analysed across three blots, and the CV for intrinsic ERK2 remained below 10%, indicating a stable assay system (Supplementary Fig. 6). This value directly reflects the inter-assay variation of the DigiWest; intra-assay variation (CV of replicate measurements from the same bead-set) was found to be below 5%. On the basis of these capabilities of the newly developed DigiWest approach, we decided to perform a comprehensive kinome analysis using a cell model for Lapatinib resistance. At the same time, an unbiased mass-spectrometry-based analysis was performed to identify differentially expressed kinases, and the data sets from the different platforms were used for cross-platform comparison.

### Kinome analysis of Lapatinib-resistant cells

**Figure 4:**
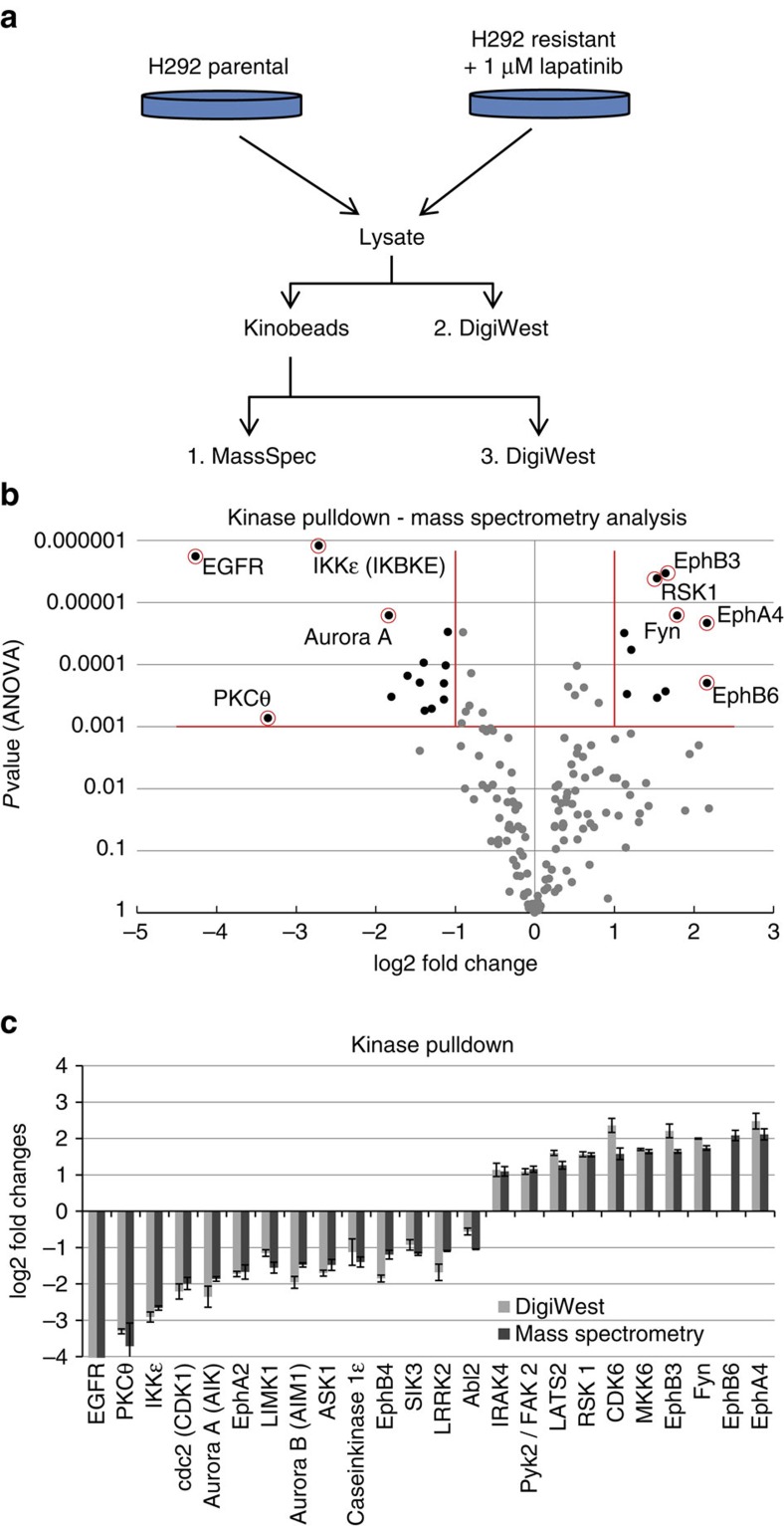
Lapatinib resistance in the H292 lung cancer cell line. (**a**) Experimental setup. Parental and Lapatinib-resistant H292 cell lines were lysed, and a portion was used for Kinobead pull-down and mass spectrometry analysis, followed by analysis using DigiWest. Whole-cell lysates were analysed using DigiWest. (**b**) Volcano plot of the mass spectrometry analysis (in triplicate), log2 fold change of the Lapatinib-resistant H292 cell line compared with the parental cell line. (**c**) A set of 24 kinases displayed significant changes (ANOVA *P*<0.001, fold change>2, peptides≥2). This set of kinases was re-analysed using DigiWest. The Pearson's correlation coefficient for these two methods was 0.98 (EGFR excluded). EphB6 could not be measured because no suitable antibody was available.

The development of resistance to kinase inhibitors presents a significant problem associated with this class of cancer therapeutics. Clinically important examples of resistance are known for Lapatinib^16^, a receptor tyrosine kinase inhibitor that specifically targets EGFR (ErbB-1) and HER2 (ErbB-2). Although Lapatinib treatment of metastatic, Her2-overexpressing tumours often results in substantial clinical responses, resistance and relapse are frequently observed^16^. To analyse changes in cellular signalling processes during the development of resistance, the mucoepidermoid pulmonary carcinoma cell line H292 was rendered Lapatinib-resistant by cultivation in the presence of increasing amounts of the drug. The resulting Lapatinib-resistant cell line had an IC_50_ of ∼800 nM in a cell-viability assay, whereas the parental line was responsive at 10 nM (see Supplementary Fig. 7 for details); no additional parallel analyses (for example, quantification of mitotic and apoptotic index, RNA profiling) were performed with these two cell lines. Although this is clearly a limitation, the proteomic profiles alone in the parental as compared with the Lapatinib-resistant cell line still yield very valuable insights into the biology of drug resistance. To detect changes in the status of signalling pathways, a chemical proteomics approach was chosen; kinase affinity pull-downs were performed in triplicate using Kinobeads^4^ on parental and Lapatinib-resistant H292 lines, and kinase-enriched fractions were analysed by mass spectrometry. In addition, all Kinobead pull-downs and initial whole-cell lysates were analysed using the DigiWest approach (Fig. 4a).

The unbiased mass-spectrometry approach identified a set of 24 kinases that showed significant differences (Lapatinib-resistant cell line versus parental H292; ANOVA *P*<0.001, fold change>2, number of peptides ≥2; triplicates; Fig. 4b,c, see Supplementary Data 2 for the full data set). Since resistant cells were cultured in the presence of 1 μM Lapatinib, the nucleotide-binding pockets of EGFR and HER2 were occupied by the inhibitor and thus these proteins were not captured by the Kinobeads resulting in an apparent underrepresentation in the resistant cell line. It concomitantly suggested that resistance is not caused by mutation of the primary targets of the inhibitor as both, HER2 and EGFR are still able to bind Lapatinib. All samples were re-analysed via the DigiWest using antibodies against differentially expressed kinases. No working antibody could be identified for one of the kinases, whereas specific peaks were detected and integrated for the remaining 23 kinases (Fig. 4c, Supplementary Fig. 8). Despite use of two different assay platforms, highly similar expression patterns were observed (Pearson's correlation coefficient=0.98).

**Figure 5:**
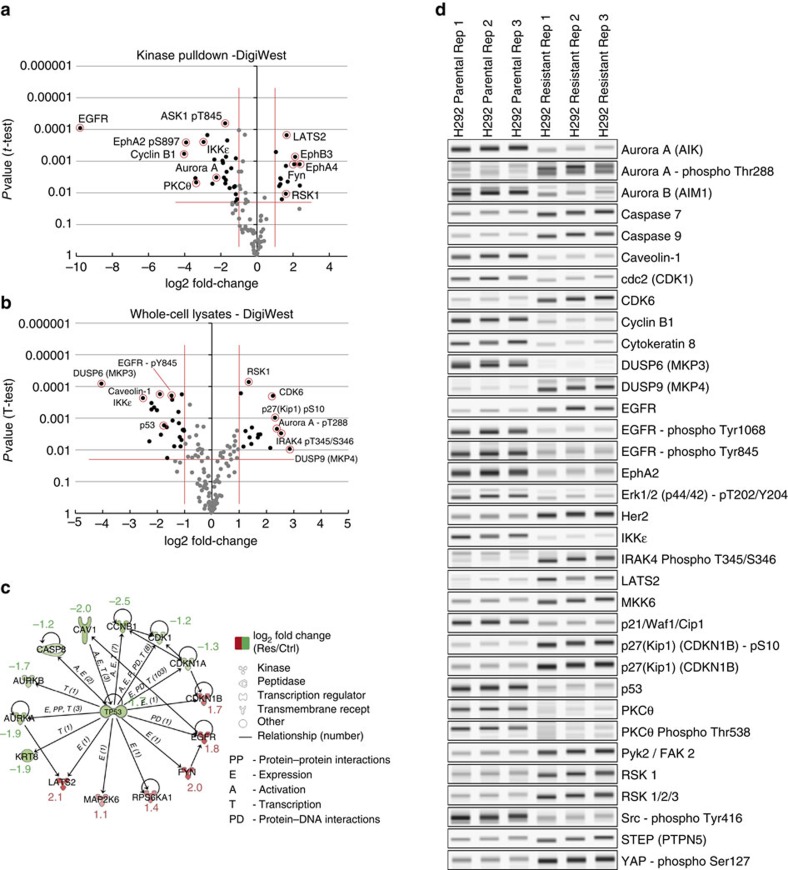
DigiWest results. (**a**) Volcano plot of the Kinobead pull-down analysis. The data contained in this volcano plot are based on quantified signals obtained from an equivalent to about 1,000 western blot lanes. Samples were performed in triplicate. A total of 185 analytes were detected, including 74 phosphorylations. Log2 fold change of the Lapatinib-resistant H292 cell line compared with the parental cell line. *P* values are from *t*-tests with Welch approximations. (**b**) The same analysis as in **a**, but for whole-cell lysates. (**c**) Detail of the Ingenuity pathway analysis indicating p53 as the central regulator. (**d**) Whole-cell lysate analysis. Digital reconstruction of western blots (greyscale maps) from the DigiWest data representing the 34 most significantly changed analytes in alphabetical order.

To obtain a broader view of the changes in cellular signalling, DigiWest analyses were performed to detect differential phosphorylation in the affected pathways. A total of 185 western blot equivalents, including the analysis of 72 site-specific phosphorylations, were performed on the kinase-enriched fractions (Fig. 5a) and whole-cell lysates (Fig. 5b). The DigiWest approach allowed therefore not only the re-analysis of the kinases found to be deregulated in the resistant state; a significant extension to phosphorylation sites present in proteins in the affected pathways was possible. In addition, regulatory proteins that are not easily detected in enriched kinase fractions were chosen for a more detailed analysis. A focus was set to protein phosphatases and transcription factors, as was on the detection of tumour markers (for examplw, cytokeratins).

Differential phosphorylation was observed for a variety of proteins; a substantial increase in phosphorylated Aurora A, p27 and IRAK4 was detected; for other proteins a decrease in phosphorylation was observed (MEK1, Src, Erk1/2, different sites in EGFR; see the complete list in Supplementary Data 3). The total amount of different regulatory proteins was found to be changed. The notable upregulation of EGFR and Her2 may represent a compensatory mechanism to escape the high doses of Lapatinib, but as the phosphorylation and therefore activity of the receptors is still strongly reduced in the resistant cell line, this was not interpreted as the major cause of resistance.

Strong differences were detected in the expression levels of Ephrin-receptors; EphA2 andEphB4 were strongly reduced in Lapatinib-resistant cells, whereas the levels of EphA4, EphB3 and EphB6 increased more than fourfold. At the same time, Serine 897 phosphorylation of EphA2 dropped to low levels, indicating a major rewiring of Ephrin signalling during the development of resistance (Fig. 5d and Supplementary Data 3). Measurements using whole-cell lysates enabled the additional detection of a variety of non-kinases. In particular, the levels of two dual-specificity phosphatases were observed to invert. DUSP6 was strongly downregulated in resistant cells, whereas DUSP9 was highly upregulated. Pathway analysis (Ingenuity Pathway Analysis software, Qiagen, Germany) was used to identify canonical pathways associated with the observed differences in expression. An regulator analysis was performed to identify the cascade of transcriptional regulators that are able to explain the observed gene expression changes. Out of the 37 differentially expressed proteins used for the analysis, 15 were associated with p53 (*P* value of 2.05E-15), which makes p53 the regulator which is by far the most significantly associated with the changes observed in the dataset (see Supplementary Fig. 9). The mechanistic network generated shows p53 and associated interactions from the data set and it does predict that p53 is inactivated (*z*-score=−0.442; Fig. 5) in the Lapatinib-resistant state. This prediction could be confirmed by performing additional DigiWest assays using antibodies targeting p53. This is in line with the observation that in case of clonal selection for resistant H292 cells loss of p53 plays an important role in resistance to EGFR inhibitors^17^.

### Protein expression analysis in primary tumour cells

**Figure 6:**
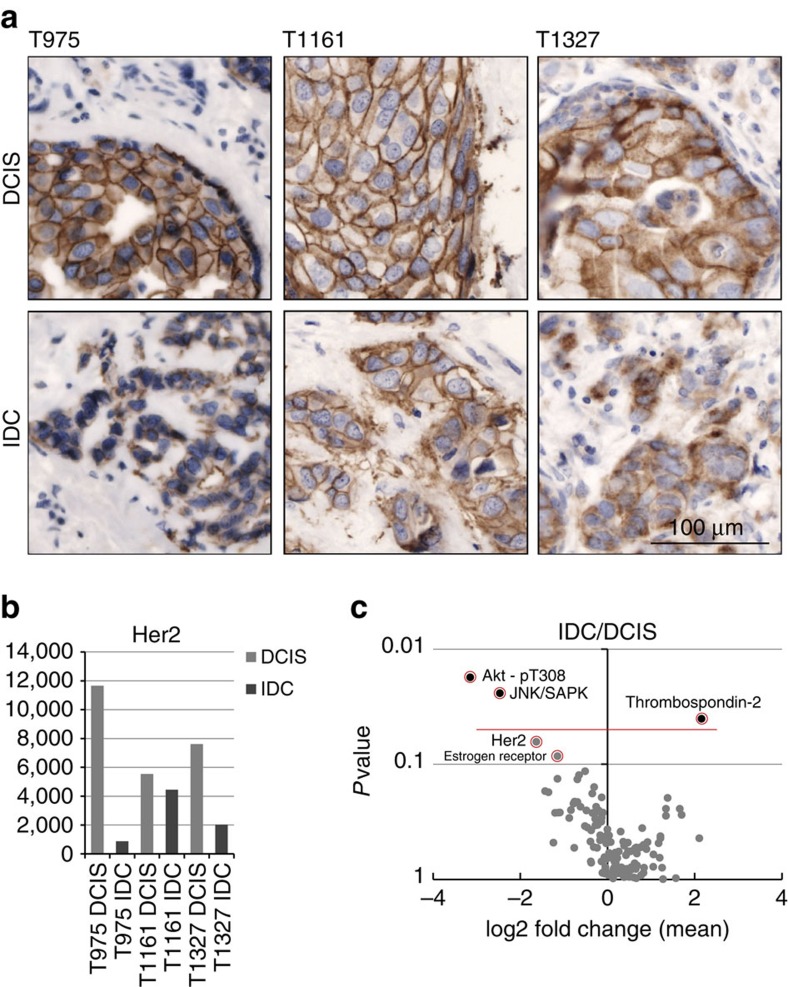
DigiWest from LCM-derived primary tumour cells. Paired IDC and DCIS LCM samples were analysed using DigiWest.(**a**) Her2-stained sections, paired IDC and DCIS from three patients. (**b**) Her2 signals for the LCM samples on DigiWest. (**c**) Volcano plot representing the mean IDC/DCIS ratio for all measured analytes versus significance. *P* values were derived from *t*-tests with Welch approximation. The data contained in this volcano plot are based on quantified signals obtained from an equivalent to about 1,000 western blot lanes.

Protein analysis of laser-capture-microdissected material is challenging because only limited amounts of protein are available for analysis. To demonstrate that the DigiWest technology is capable of dealing with such scarce source material, primary tumour cells were collected from mammary carcinoma tissue via laser-capture microdissection, and DigiWest protein profiling was performed to detect differences between histologically different tumour types. Cryosections containing invasive ductal carcinoma (IDC) and ductal carcinoma *in situ* (DCIS) were selected, and a few thousand cells from each tumour type were collected from successive sections, lysed and subject to the DigiWest. Although the amount of protein obtained was low (<5 μg), nearly 200 western blot equivalents were performed on the matched samples; ∼20 ng of protein was required for each antibody-based analysis. The chosen antibodies cover major signalling pathways known to play a role in invasiveness and carcinogenesis. Because the three matched tumour pairs that were analysed had been stained for the presence of Her2 during routine histology, the signal intensity as determined by DigiWest could be compared with the immunohistological pictures (Fig. 6a,b). The intensity detected via histology was recapitulated by the DigiWest, indicating the comparability of these two approaches. Using 189 different antibodies (including 67 phosphorylation-specific binders), the signalling status of the matched DCIS-IDC tumour pairs was analysed. This protein profiling approach aimed at detecting changes that occur during the transition from a non-invasive to an invasive tumour. Proteins that exhibited changes in expression between DCIS to IDC were identified (Fig. 6c, Supplementary Data 4).

Differences were observed for the known tumour markers Thrombospondin-2, Estrogen-receptor and Her2, and the levels of the central regulatory JNK/SAPK kinase decreased. AKT phosphorylation at Thr308, which is indicative of pathway activation^18^, was lower in all analysed IDC samples. Thus, the DigiWest approach displays high sensitivity is capable of providing high-resolution protein expression data, even from limited material.

## Discussion

With thousands of working antibodies available, western blot-based approaches for systems biology would be highly useful, but material consumption and workload make such an approach impractical. DigiWest represents a high-throughput version of the classical western blot method that uses a bead-based microarray platform for signal generation. The sensitivity, signal linearity and reproducibility of DigiWest are comparable to the best available western blot systems, and the linear range of the obtained signal spans more than three orders of magnitude. This adaptation of the protein blot to a widely used bead-based microarray platform allows hundreds of antibody-based assays to be run in a microtiter plate format. The result is a ‘liquid Western blot' that is amenable to automation and leads to substantial output.

DigiWest is not the only approach capable of bringing antibody-based protein expression and modification analysis to systems biology. RPPAs are well-established systems for generating complex protein expression patterns from limited amounts of sample. The main drawback is signal specificity because protein separation is not part of the workflow and because unspecific antibody binding often contributes substantial signal to the measurements. The only way to avoid this problem and obtain reliable results is to find highly specific antibodies, which are often not available. Several systems have been developed that provide information on the molecular weights of the detected molecules. The Micro-Western Array^19^ a miniaturized version of the classical western blot, and the capillary western system^20^ commercialized by Proteinsimple are the most advanced technologies, whereas the recently developed ‘Single Cell Western'^21^ allows the analysis of very low amounts of sample. Although these systems resemble the western blot workflow, they do not provide the spatial resolution of the classical western blot or the bead-based system described here. The Micro-Western can be run as an automated system in which sample application, protein separation for dozens to hundreds of samples is possible, bringing the throughput that is possible with RPPA into reach. Nevertheless, no commercial Micro-Western system is available and in contrast to the DigiWest the setup of such a system not only requires expert knowledge on protein microarraying^22^, but also a substantial investment. The combination of standard western blotting equipment with the widely distributed Luminex technology (10.000+ installed machines) allows easy implementation of the DigiWest approach in a standard protein laboratory. The strength of the newly developed system is therefore not the protein profiling of thousands of samples for a small set of proteins, but in the possibility to analyse hundreds of samples for hundreds of proteins or for relevant modifications. Thereby it presents a focused proteomics approach that is especially suited for detecting changes in regulatory protein networks. In this respect it shares some of the characteristics of newly developed mass–spectrometry-based analysis approaches like MRM^23^ or immune-affinity MS based systems^24^,^25^, that allow focused and thereby usually hypothesis-driven experimental approaches. The bead-based western approach presents a viable alternative to this type of analysis, since cost of the system is comparatively low and only a minute amount of sample is required for analysis.

While the similarity of the DigiWest to the classical western blot has to be seen as a strong point, it also points to obvious weaknesses. Reproducibility and interpretation of western blot data is known to be critical and for obtaining reliable results a defined workflow is required. This is indeed a crucial point for our system and a strictly controlled process is required when implementing the system. Second, the method is a capable tool for discovery studies of dozens to hundreds of samples, but so far not optimal for higher sample throughput, but we are planning to overcome this through technical advancement. Third, the nature of this platform does allow for semi-quantitative analyses and for absolute quantification of individual analytes, but it is inevitably not suitable for absolute quantification of hundreds of protein analytes. While we show that data acquisition on the Luminex platform as well as peak identification and integration can be carried out with good reproducibility using a relatively simple workflow, irregularities in the sample composition may lead to difficulties in identifying corresponding peaks. Finally, DigiWest is dependent on the availability of validated antibodies, which limits its discovery potential to proteins and translational modifications where such antibodies are available; as the number of validated antibodies is steadily increasing, this seems to be a question of time. The main advantage of DigiWest, namely the capability to analyse hundreds of proteins and protein modifications in parallel, does in our opinion clearly outbalance these limitations.

The generation of more than 200 western blot equivalents from primary human tumour cells collected by laser microdissection demonstrates that DigiWest can also be used with limited material; <25 cells were calculated to be required for one western blot equivalent. One of the strengths of the DigiWest system is the ability to run focused proteomics studies to detect differences in the activation states of cellular signalling cascades. The analysis of a H292 lung cancer cell line rendered resistant to Lapatinib offers an example of how DigiWest can be used. The unbiased mass spectrometry approach that allowed the *de novo* identification of changes in the kinome was followed by a focused re-analysis of the samples by DigiWest. This led to conformation of the initial observations and in addition changes in the phosphorylation state of relevant kinases were detected; the antibody-based analysis of whole-cell lysates permitted the detection of differentially expressed regulatory proteins that were not easily visible in the mass-spectrometry-based system. Only the combination of both technologies resulted in the identification of a role of p53-regulated processes in Lapatinib resistance. Although DigiWest requires usable antibodies, measurements can be performed with crude cell extracts, making the approach viable for the focused analyses of limited unpurified samples. Clinical material such as primary human tumour cells collected from histological sections might be of particular interest because large collections of clinical specimen have been collected in biobanks which allow a lot of retrospective analysis to be done on these samples. Differences in the amounts of low-abundance proteins and in the phosphorylation states of signalling proteins can be detected with reasonable throughput and high reliability. Given the recent availability of antibody collections that cover most of the human proteome and the many phosphorylation-specific binders, comprehensive cellular signal transduction analyses are now possible. Transferring the western blot methodology to beads creates an analysis tool that retains the advantages of this classical protein detection method while adding the ability to apply hundreds of different antibodies in a rapid and sensitive assay system.

## Methods

### Cell culture and lysate preparation

The human hepatoblastoma cell line HepG2 (American Type Culture Collection, Manassas, VA, USA) was cultured in α-minimum essential medium (Life Technologies, Darmstadt, Germany) supplemented with 10% fetal calf serum, and cells were maintained at 37 °C in a humidified atmosphere of 5% CO2/air. On reaching 80–90% confluency, the cells were washed with phosphate-buffered saline (PBS) before being collected and stored at −80 °C. Cell lysates were generated from frozen cell pellets under denaturing conditions by the addition of a 10-fold excess (v/w) of CeLyA Lysis Buffer CLB1 (Zeptosens—a Division of Bayer, Leverkusen, Germany). For denaturing gel electrophoresis, the obtained lysates were diluted in LDS sample buffer (Life Technologies) according to the manufacturer's instructions and denatured by heating for 10 min at 70 °C.

### Resistant cell line generation and cell culture and lysis

NCI-H292 cells (American Type Culture Collection) were cultured in IMDM (Iscove's Modified Dulbecco's Medium, PAA, Pasching, Austria) supplemented with 10% (v/v) fetal bovine serum (PAA) and 1% (v/v) antibiotic/antimycotic solution (PAA) at 37 °C in humidified air and 10% CO2. To induce resistance, H292 cells were gradually exposed to increasing concentrations of Lapatinib (LC Laboratories, Woburn, MA, USA) at intervals of 8–14 days (Supplementary Fig. 7). After 6 months, cells growing in the presence of 1 μM Lapatinib were continuously maintained in culture. The parental, sensitive cell line was cultured in parallel over the same period of time. On reaching 80–90% confluency, the cells were washed twice with ice-cold PBS and lysed with 1 × compound pull-down (CP) buffer (50 mM Tris/HCl pH 7.5, 5% Glycerol, 1.5 mM MgCl_2_, 150 mM NaCl, 1 mM Na3VO4, 25 mM NaF, 0.8% NP-40) containing protease (Roche Applied Science, Mannheim, Germany) and phosphatase inhibitors (Sigma-Aldrich, Munich, Germany). Lysed cells were clarified by centrifugation at 150,000*g* for 1 h at 4 °C. Protein concentrations were determined using the Coomassie (Bradford) protein assay kit (Thermo Fisher Scientific, Schwerte, Germany). All samples were stored at −80 °C until further use.

### Cell viability

Cells were seeded in complete culture medium in 96-well plates at a concentration of 5 × 10^3^ cells per well and allowed to adhere overnight. The next day, cells were exposed to increasing concentrations of Lapatinib or 0.1% DMSO (vehicle control) for 72 h at 37 °C in 10% CO_2_. The XTT Cell Proliferation Kit II (Roche Applied Science) was used to measure cell viability according to manufacturer's instructions. GraphPad Prism v.5.01 was used to derive curve fits using a nonlinear regression model with a sigmoidal dose response.

### Kinobead affinity purification

Kinobead pull-downs from control and resistant cell lysates were performed in triplicate as previously described^26^,^27^. Briefly, cell lysates corresponding to 10 mg protein were diluted with equal volumes of 1 × CP buffer to reduce the detergent concentration from 0.8% to 0.4% NP-40 and cleared by ultracentrifugation at 150,000*g* and 4 °C for 20 min. A total of 100 μl (settled volume) of in-house synthesized Kinobeads were incubated with the cleared lysates in a head-over-end tube rotator for 1 h at 4 °C. After several washing steps, bound proteins were eluted with 2 × NuPAGE LDS Sample Buffer (Life Technologies) containing 50 mM DTT. Eluates were reduced by incubating for 30 min at 50 °C and subsequently alkylated with 55 mM IAA for 30 min at room temperature in the dark. After desalting and concentration by a short gel electrophoresis on 4–12% NuPAGE gels (Life Technologies), stained bands were excised and subjected to in-gel digestion using trypsin (Promega, Mannheim, Germany). Samples were dried in a vacuum concentrator and stored at −20 °C until LC-MS/MS analysis.

### Tissue preparation

The cryo-preserved breast cancer tissues were selected from the cryo tissue bank of the Department of Obstetrics and Gynecology of the University Hospital, Tubingen, Germany. None of the patients included had received preoperative systemic treatment. Areas of DCIS and IDC were selected on the basis of repeated evaluation of high-quality hematoxylin and eosin (H&E)-stained sections by a pathologist. Specimens were obtained with informed consent from patients who were treated at the Department of Obstetrics and Gynecology of the University Hospital, Tubingen, Germany (ethical consent of the Medical Faculty, Tubingen: AZ 266/98).

The tissue samples were fixed in OCT Compound (Sakura Finetek), and serial sections were cut at 8 μm (Leica CM 3050S, Leica Microsystems, Wetzlar, Germany) on special slides (SuperFrost Plus, Langenbrinck, Emmendingen, Germany). The sections were stained with hematoxylin (Roth, Karlsruhe, Germany) for 40 s and washed in water. After a short dip in Eosin (Roth, Karlsruhe, Germany), the slides were washed again with water, followed by incubation in 70% ethanol (3 min), 2 × 100% ethanol (3 min each) and xylene (3 min).

### Laser-capture microdissection

Areas of DCIS and IDC were laser microdissected using the PixCell IIe LCM System (Arcturus, Life Technologies). The CapSure LCM Cap (Arcturus, Life Technologies) was placed directly onto the dried tissue slide. Cells of interest were shot with an infrared laser (wavelength 980–1,064 nm) and pressed to the membrane of the cap. The caps were stored at −20 °C until further use.

### LCM sample preparation

Laser-capture microdissection (LCM) samples were lysed as described^28^ in a 1:1 mixture of 2 × LDS sample buffer containing 2 × reducing agent (Life Technologies) and TPER (Pierce). LCM caps corresponding to identical samples were lysed sequentially by placing them in a 0.5- ml vial (Eppendorf, Hamburg, Germany) containing 20 μl lysis buffer. The vials were flipped, placed on a Thermo Mixer Comfort (Eppendorf) and agitated for 20 min in interval mode (10 s on, 10 s off) at 1,200 r.p.m., 25 °C. The Vials were centrifuged for 1 min at 10,000 r.c.f., and the next LCM-cap was placed on the vial. After all caps were lysed, the lysates were heated to 90 °C for 20 min.

Protein determination was performed by in-gel staining. Briefly, 1 μl of each lysate was diluted by adding 10 μl lysis buffer, and 10 μl thereof was loaded onto a NuPAGE gel. A tumour tissue lysate with a known concentration was also loaded onto the gel as a standard. After electrophoresis, the gel was stained with Krypton protein stain (Thermo Scientific, Schwerte, Germany) according to the manufacturer's instructions.

### Quantification of ERK2

To quantify the cellular concentration of ERK2 in HepG2 cell lysates, a spike-in experiment was performed; defined amounts (20 ng, 5 ng, 1.25 ng, 0.31 ng, 78 pg or 19.5 pg) of recombinant human GST-ERK2 (Cell Signaling Technology) were added to 10 μg of HepG2 lysate in LDS lysis buffer, and triplicate samples were loaded onto NuPAGE SDS-PAGE gels for analysis. For both DigiWest analysis and classical western blotting, three replicates of each dilution were used.

### Gel electrophoresis and blotting for western blot

For gel electrophoresis and blotting, the commercial NuPAGE SDS-PAGE gel system (Life Technologies) was used. Proteins were separated using 4–12% Bis-Tris gels in NuPAGE MES running buffer according to the manufacturer's instructions. Proteins were blotted onto nitrocellulose (GE Healthcare, Freiburg, Germany) membranes according to the instructions. Blots were blocked in 3% BSA (HepG2 blots used for comparison with DigiWest) or milk powder (GST-ERK2 spike-in experiment) in TBST, and antibodies were incubated overnight at 4 °C at the recommended dilution in 1% BSA or milk powder in TBST and washed 5 times for 5 min in TBST before fluorescently labelled secondary antibodies were added. After a 1-h incubation at room temperature, the blots were washed 5 times for 5 min. For detection on the LI-COR Odyssey (LI-COR, Bad Homburg, Germany), a donkey anti-Rabbit IgG (H+L) conjugated to IRDye 800CW (LI-COR) secondary antibody was used. For the chemiluminescent readout, HRP-labelled secondary antibodies (Dianova, Hamburg, Germany) were used. The Super Signal West Pico (Thermo Scientific) substrate was used according to the manufacturer's instructions.

Images of western blots for comparison with DigiWest were selected from a pool of western blots performed during the validation of the large antibody collection. The blots were generated by a modification of the common western blot technique. Briefly, 400 μg of HepG2 lysate in LDS sample buffer (Life Technologies) was loaded onto NuPAGE 2D-well gels, and proteins were blotted onto nitrocellulose membranes. The 60-mm-wide lanes on the blots were cut into 1.5-mm wide vertical stripes that were then incubated with the antibodies diluted in 1% BSA in TBST. Chemiluminescent detection was performed on an LAS-4000 mini (Fujifilm, Düsseldorf, Germany) or in some cases on a Kodak Image Station 440 CF (Kodak Eastman, Rochester, NY, USA). When required, western blot images were quantified using MultiGauge v3.0 (Fujifilm) software.

### Gel electrophoresis and blotting for DigiWest

The commercial NuPAGE SDS-PAGE gel system (Life Technologies) was used for protein separation and blotting. Proteins were separated using 4−12% Bis-Tris gels in NuPAGE running buffer according to the manufacturer's instructions. Proteins were blotted onto PVDF membranes (Millipore) using the described standard conditions. Blots were incubated in PBS containing 0.1% Tween-20 (PBST) for 15 min, stained with Ponceau-S for 5 min and imaged on an LAS-4000 mini (Fujifilm). The blots were then washed in PBST for 30 min with several rounds of buffer exchange and gentle agitation.

### Bead coating and preparation

The coating of magnetic MagPlex microspheres (Luminex, Oosterhout, The Netherlands) with Neutravidin (Thermo Fisher Scientific) was performed on a King Fisher 96 (Thermo Fisher Scientific) magnetic particle processor essentially as described in ref. 29. In total, 384 different individual Neutravidin-coated MagPlex (Luminex) bead-sets (∼5 × 10^6^ beads per set) were generated and transferred to storage buffer (1% BSA, 0.05% sodium azide, 0.05% Tween-20 in PBS) with a bead number adjusted to 4,000 beads per μl. For use in the DigiWest procedure, aliquots containing 20,000 or 40,000 beads per well of each bead-set were used.

### DigiWest

After protein electrophoresis and blotting, the membranes were submerged in 20 ml Biotinylation-buffer (freshly prepared 50 μM solution of NHS-PEG12-Biotin (Thermo Scientific) in PBST and incubated with gentile agitation for 1 h at room temperature in the dark to biotinylate the immobilized proteins. Excess NHS-PEG12-Biotin was removed by washing the blots three times for 1 min in PBST. After the blots were dried, each lane was cut into a comb-like structure containing 96 molecular weight fractions with a height of 0.5 mm each using a Silhouette SD electronic cutting tool (Silhouette America, West Orem, UT, USA). While the automated cutting process uses a high-resolution cutting plotter, the generated membrane strips (0.5 mm × 6 mm) were transferred manually to separate wells of a 96-well plate. Individual strips (0.5 mm × 6 mm) were placed in separate wells of a 96-well plate, and proteins were eluted by incubation in 10 μl of elution buffer^30^ (8 M urea, 1% Triton-X100 in 100 mM Tris-HCl, pH 9.5) for 90 min at room temperature, with shaking (1,200 r.p.m.). Eluted proteins were diluted by adding 90 μl of dilution buffer (5% BSA in PBS, 0.02% sodium azide, 0.05% Tween-20) per well before the 96 different Neutravidin-coated Luminex bead-sets were transferred to the plate. For 100 antibody incubations, 20,000 beads per well were added; 40,000 beads per well were added for 200 antibody incubations. Overnight incubation (room temperature at 750 r.p.m.) was performed to immobilize the biotinylated proteins on the beads. The remaining free biotin-binding sites were blocked by adding 5 μl of PEG12-Biotin (∼500 μM). The Luminex beads were pooled into bead pools (96, 192 or 384 different bead-sets containing 1, 2 or 4 complete western blot lanes), washed and stored in storage buffer (1% BSA, 0.05% Tween-20, 0.05% sodium azide in PBS) at 4 °C.

For antibody incubation, an aliquot of the bead pool containing 200 beads of each bead population was transferred into an assay plate and washed, and 30 μl of diluted antibody in assay buffer (Blocking Reagent for ELISA (Roche, Rotkreuz, Switzerland) supplemented with 0.2% milk powder, 0.05% Tween-20 and 0.02% sodium azide) was added per well; the complete list of used antibodies is found in Supplementary Table 1. Incubation with primary antibodies was performed overnight at 15 °C, 750 r.p.m. on a Thermomixer comfort (Eppendorf, Hamburg, Germany). Beads were washed twice with 100 μl of PBST before the addition of species-specific PE-labelled secondary antibody (Jackson Dianova, Hamburg, Germany) in 30 μl of assay buffer, followed by incubation for 1 h at 23 °C, 750 r.p.m. After 2 washing steps with 100 μl of PBST, detection was performed on a FlexMAP 3D instrument (Luminex).

### LC-MS/MS

Mass spectrometry was performed by coupling an Eksigent nanoLC-Ultra 1D+ (Eksigent, Dublin, CA, USA) to an Orbitrap Velos instrument (Thermo Scientific). Peptides were delivered to a trap column (100 μm × 2 cm, packed in-house with Reprosil-Pur C_18_-AQ 5 μm resin, Dr Maisch, Ammerbuch, Germany) at a flow-rate of 5 μl min^−1^ in 100% solvent A (0.1% formic acid (FA), in HPLC-grade water). After 10 min of loading and washing, peptides were transferred to an analytical column (75 μm × 40 cm, packed in-house with Reprosil-Gold C_18_, 3 μm resin, Dr Maisch, Ammerbuch, Germany) and separated using a 210-min linear gradient of solvent B (0.1% FA in acetonitrile) from 7 to 35% at a flow-rate of 300 nl min^−1^. The Orbitrap Velos instrument was operated in data-dependent acquisition mode, automatically switching between MS1 and MS2. Full-scan MS1 spectra (m/z 300–1,300) were acquired in the Orbitrap at 30,000 resolution (m/z 400) using an automatic gain control target value of 1e6 and a maximum ion injection time of 250 ms. Internal calibration was performed using (Si(CH3)2O)6H+ (m/z 445.120025)) present in ambient laboratory air. High-resolution HCD MS/MS spectra were generated for the ten most abundant precursor ions (automatic gain control target value 3e4, normalized collision energy of 40%) and analysed in the Orbitrap at a resolution of 7,500 (m/z 400). Precursor ion isolation width was set to 2.0 m/z, and the maximum injection time for MS2 was 500 ms.

### Peptide identification and quantification

Intensity-based label-free quantification using Progenesis LC-MS v.3.1 (Nonlinear Dynamics, Newcastle, UK) was performed essentially as described^31^. Briefly, after the selection of one sample as a reference, the retention times of all eluting precursor m/z values were aligned to generate a large list of ‘features' representing the same peptide in each sample. Features with <2 or >9 charges, as well as features with two or fewer isotopes, were excluded. After alignment and feature filtering, replicate samples were grouped together, and the raw abundances of all features were normalized. The MS/MS spectra for each feature were transformed into peak lists and exported to generate Mascot generic files. The Mascot generic files were searched against the protein sequence database IPI human (v.3.68, 87,061 sequences) using Mascot (v.2.4.1, Matrix Science, London, UK). The following search parameters were applied: carbamidomethylation of cysteine residues as fixed modification; serine, threonine, tyrosine phosphorylation and methionine oxidation as variable modifications. Trypsin was specified as the proteolytic enzyme, and up to two missed cleavages were allowed. The mass tolerance was set to 7 p.p.m. for precursor ions and 0.02 Da for fragment ions. After percolation, the search results were exported in ‘.xml' format and matched to the features identified with Progenesis. For protein quantification, the feature intensities of all unique peptides of a protein were summed. Only kinases quantified with 2 or more unique peptides were considered for further analysis. To address statistical significance, *P* values were calculated using analysis of variance. Protein–protein interactions, regulators and pathway associations were derived using Ingenuity pathway analysis (IPA, Qiagen, Hilden, Germany).

### Ingenuity pathway analysis

All proteins changing in the DigiWest analysis of whole-cell lysates and/or Kinobead pull-downs (arbitrary log_2_ FC cutoff >1/<−1) were imported (together with the respective log_2_ fold change) into the Ingenuity pathway analysis software.

The specific settings were as follows: only direct relations are considered. Only experimentally observed and high confidence predictions were allowed. Allowed datasources for interactions included only curated Ingenuity Expert Information (Ingenuity Expert findings and Ingenuity ExpertAssist Findings) and protein–protein interactions (BIND, Cognia, DIP, Interactome studies, MINT, MIPS). Species: human. Tissue & Cell Lines: All. Mutation: All.

Top canonical pathways associated with the uploaded data set were extracted (see Supplementary Fig. 9). Next, an regulator analysis was performed to identify the cascade of transcriptional regulators that are able to explain the observed gene expression changes in our data set. Out of 37 input proteins, 15 were associated with p53 (*P* value of 2.05E−15), which makes p53 the regulator that is by far the most significantly associated with the changes, observed in the data set (see Supplementary Fig. 9). The meachanistic network showing p53 and its associated interactions with proteins regulated in the data set only contains directed edges showing the association type (activation, protein–protein interaction and so on), the number of curated observations/relations is indicated in brackets beside the respective edge, the colour-coded IPA predicitions are left out (see Fig. 5c). On the basis of the mechanistic network, p53 is predicted to be inactivated (*z*-score=−0.442), which was later on confirmed by the DigiWest measurements.

### Immunohistochemistry

Formalin-fixed, paraffin-embedded tissue blocks corresponding to the aforementioned tumour samples were retrieved from the archives of the Institute of Pathology at Tubingen University. All immunohistochemical stains were performed on a Ventana BenchMark XT (Ventana Medical Systems, Tucson, Arizona) automated stainer according to the following protocol: deparaffinized, 3-μm-thick sections were mounted on charged slides and subjected to pre-treatment with cell conditioning solution CC1 (Ventana Medical Systems) at 37° for 1 h. Slides were incubated with 1:1,250 diluted Her2-specific antibody (Polyclonal Rabbit Anti-Human c-erbB-2 Oncoprotein, A0458, Dako, Glostrup, Denmark) for 32 min at 37 °C, followed by a biotinylated detection kit (iViewDAB-Detection-Kit; Ventana Medical Systems) according to the manufacturer's protocol. Slides were counterstained with hematoxylin and bluing reagent, washed and dehydrated with an ascending alcohol series, and then covered with Cytoseal.

### DigiWest data analysis

An Excel-based (Microsoft) analysis tool was used for data analysis. The Luminex data were represented as graphs. Each graph was composed of 96 values derived from the 96 molecular weight fractions from one sample and antibody incubation. In the first step, the bead background and secondary antibody-specific signal (blank) were subtracted. The positions of the specific peaks from a collection of 8 to 10 proteins covering the molecular weight range from 15 kDa to 250 kDa were used to calculate a molecular weight marker that assigned a molecular weight to each of the 96 molecular weight fractions. XLfit (IDBS, London, UK) was used for fitting and extrapolating the molecular weights.

This marker was used for the selection of analyte-specific peaks. The molecular weight of an analyte was provided, and the algorithm searched for an adjacent peak (local maximum). A peak was defined by a decrease in the adjacent values. The algorithm defined the borders of the peak and calculated a baseline based on the local background. Thus, the molecular weight fractions to the left and right for the peak were used. The area of the peak over baseline was integrated. Altogether with some built-in options, the algorithm was optimized to select all peak-like structures, and along with some options to influence the peak selection process when necessary. Statistical analysis of DigiWest data were performed using the MEV 4.8.1 (ref. 32) software package.

### Graphical data representation

DigiWest data are composed of 96 values derived from the 96 molecular weight fractions for each sample and antibody incubation and were used to create images that mimic western blot images. Therefore, the background-subtracted Luminex data were used. For each analyte, the values from all samples were normalized to values from 0 1. These normalized data were loaded into the MEV 4.8.1 (ref. 32) software package, and a heatmap was generated by applying a greyscale colour scheme from 0 (white) to 1 (black). The heat-maps were saved as images and transferred to Photoshop (Adobe, San Jose, CA, USA), wherein a Gaussian diffusion filter was applied to blur the images. The radius was set to half the element height.

### Data availability

The authors declare that the data supporting the findings of this study are available within the article and its Supplementary Information files. Any additional relevant information is available from the corresponding author on request. Source data for (Figs 3, 4, 5) are provided in the Supplementary Information files.

## Additional information

**How to cite this article**: Treindl, F. *et al*. A bead-based Western for high-throughput cellular signal transduction analyses. *Nat. Commun.* 7:12852 doi: 10.1038/ncomms12852 (2016).

## Supplementary Material

Supplementary Figures Supplementary Figures 1-9

Supplementary Data 1 List of employed antibodies

Supplementary Data 2 Protein and peptide quantification and identification data for the mucoepidermoid pulmonary carcinoma cell line H292 and its Lapatinibresistent derivative

Supplementary Data 3 Protein expression values determined by DigiWest for the mucoepidermoid pulmonary carcinoma cell line H292 and its Lapatinibresistent derivative

Supplementary Data 4 Protein expression values for determined by DigiWest for primary IDC and DCIS cells
