# Supplementary material for: A bead-based western for high-throughput cellular signal transduction analyses
Source: Nat Commun. 2016 Sep 23;7:12852. doi: 10.1038/ncomms12852 (PMC5036152; doi:10.1038/ncomms12852)
Supplement: Supplementary Figures — 1-9 [file ncomms12852-s1.pdf]

Supplementary figure 1: Comparison of DigiWest and Western blot.

20 µg of HepG2 lysate were processed using the described DigiWest procedure and aliquots of the generated bead pools were incubated with 104 commercially available antibodies; for comparison the same set of antibodies was used in standard Western blotting. Classical Western blot (left), DigiWest data, illustrated as a graph (middle) and grayscale maps (generated from numerical DigiWest data) are shown side by side for all tested antibodies. The antibodies in this set were selected to cover characteristics relevant for Western blotting: (i) single band antibodies and antibodies recognising multiple bands (ii) antibodies that result in high and in low signal, (iii) antibodies that detect proteins over the entire molecular weight range of the employed gel system, (iv) monoclonal and polyclonal antibodies and (v) antibodies recognizing soluble and membrane bound proteins. All Western blots we show in supplementary figure 1 were performed on one batch of HepG2 cells. N.B.: While Western blotting conditions and image acquisition for all these antibodies occurred under identical conditions, the employed protocol varies slightly from the described DigiWest workflow. Differences in buffer composition and a different blotting membrane (see methods section) contribute to the small differences seen in this comparison.

Supplementary figure 1

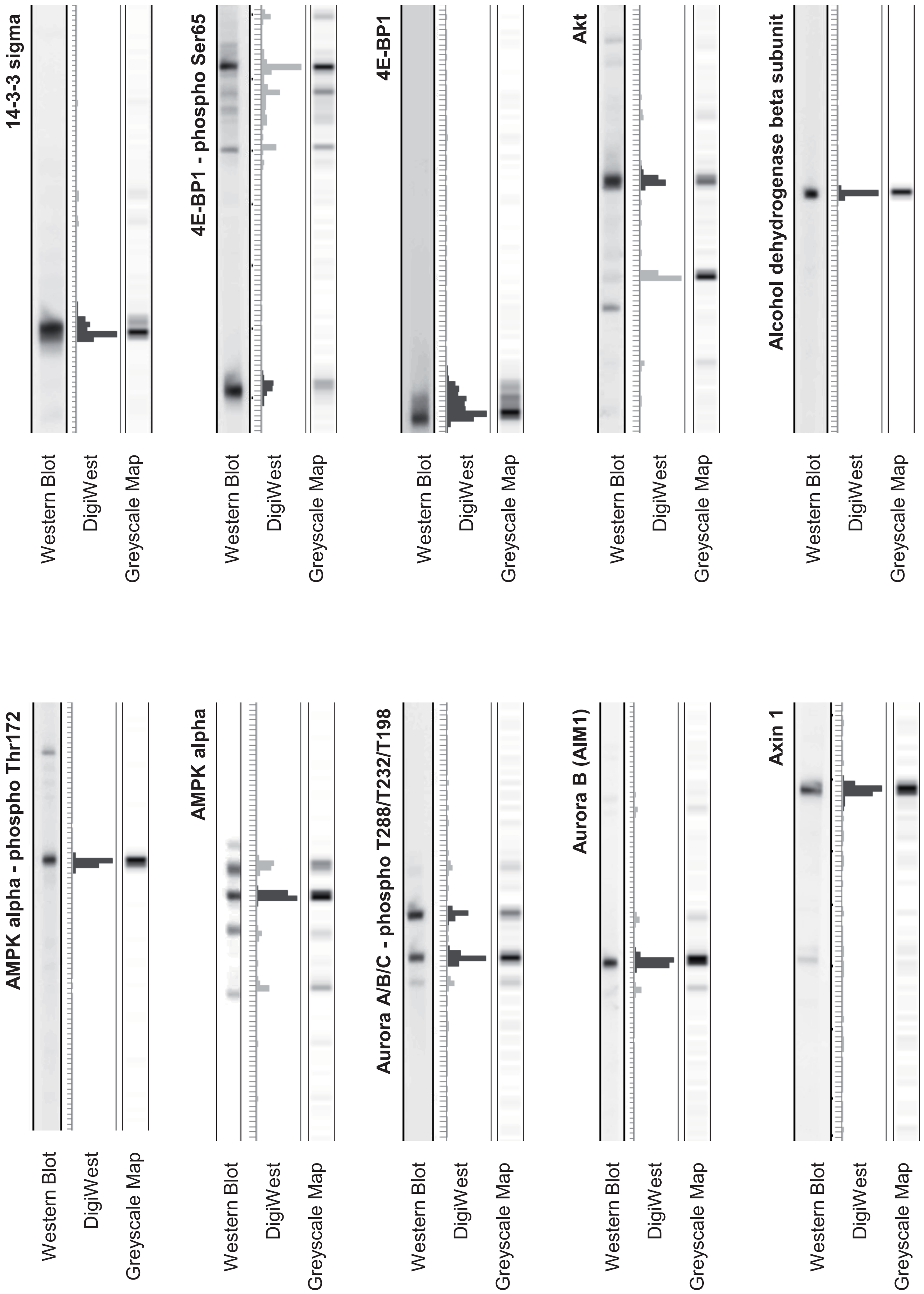

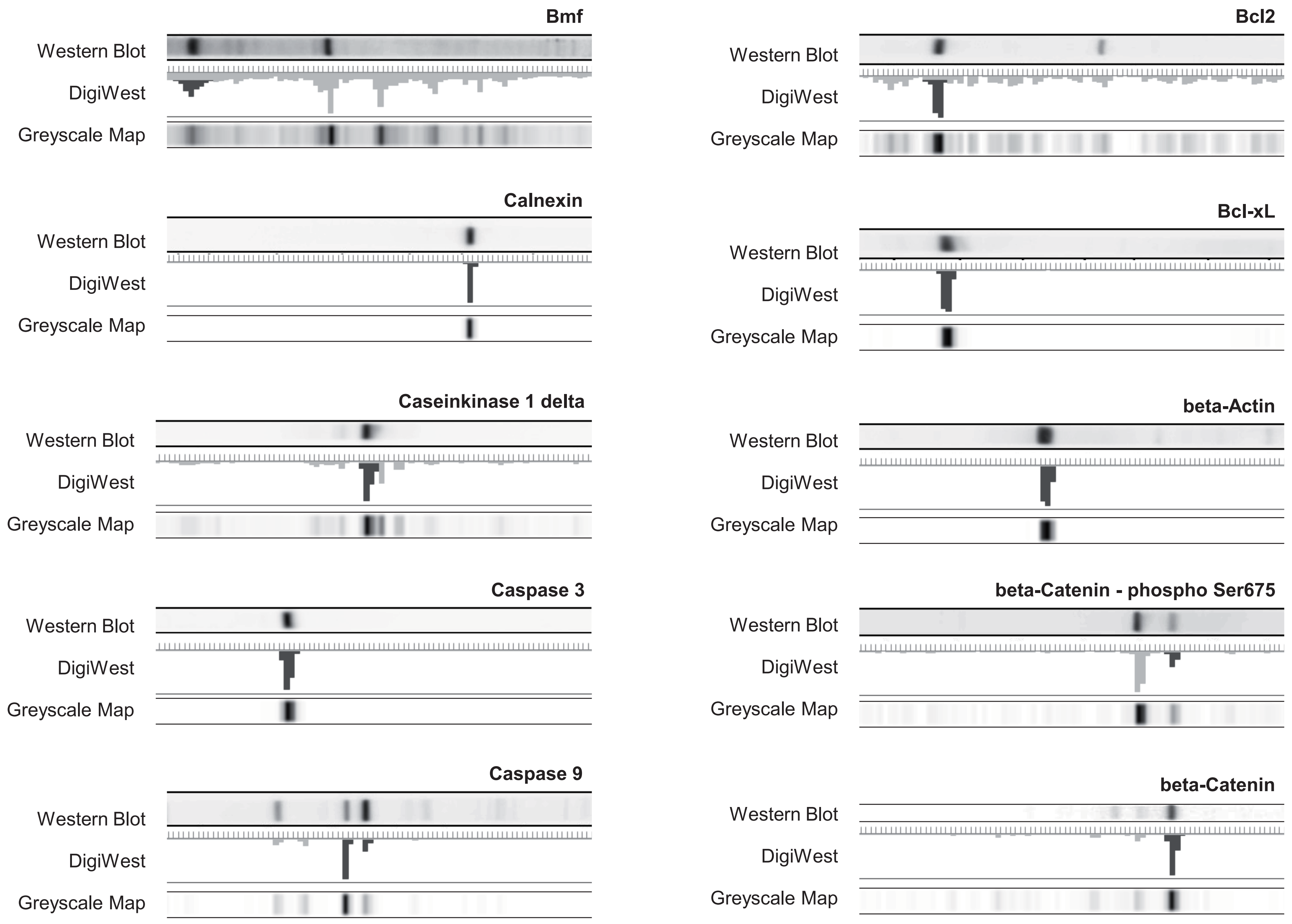
Bmf
Western Blot
DigiWest
Greyscale Map
Calnexin
Western Blot
DigiWest
Greyscale Map
Caseinkinase 1 delta
Western Blot
DigiWest
Greyscale Map
Caspase 3
Western Blot
DigiWest
Greyscale Map
Caspase 9
Western Blot
DigiWest
Greyscale Map
Bcl2
Western Blot
DigiWest
Greyscale Map
Bcl-xL
Western Blot
DigiWest
Greyscale Map
beta-Actin
Western Blot
DigiWest
Greyscale Map
beta-Catenin - phospho Ser675
Western Blot
DigiWest
Greyscale Map
beta-Catenin
Western Blot
DigiWest
Greyscale Map

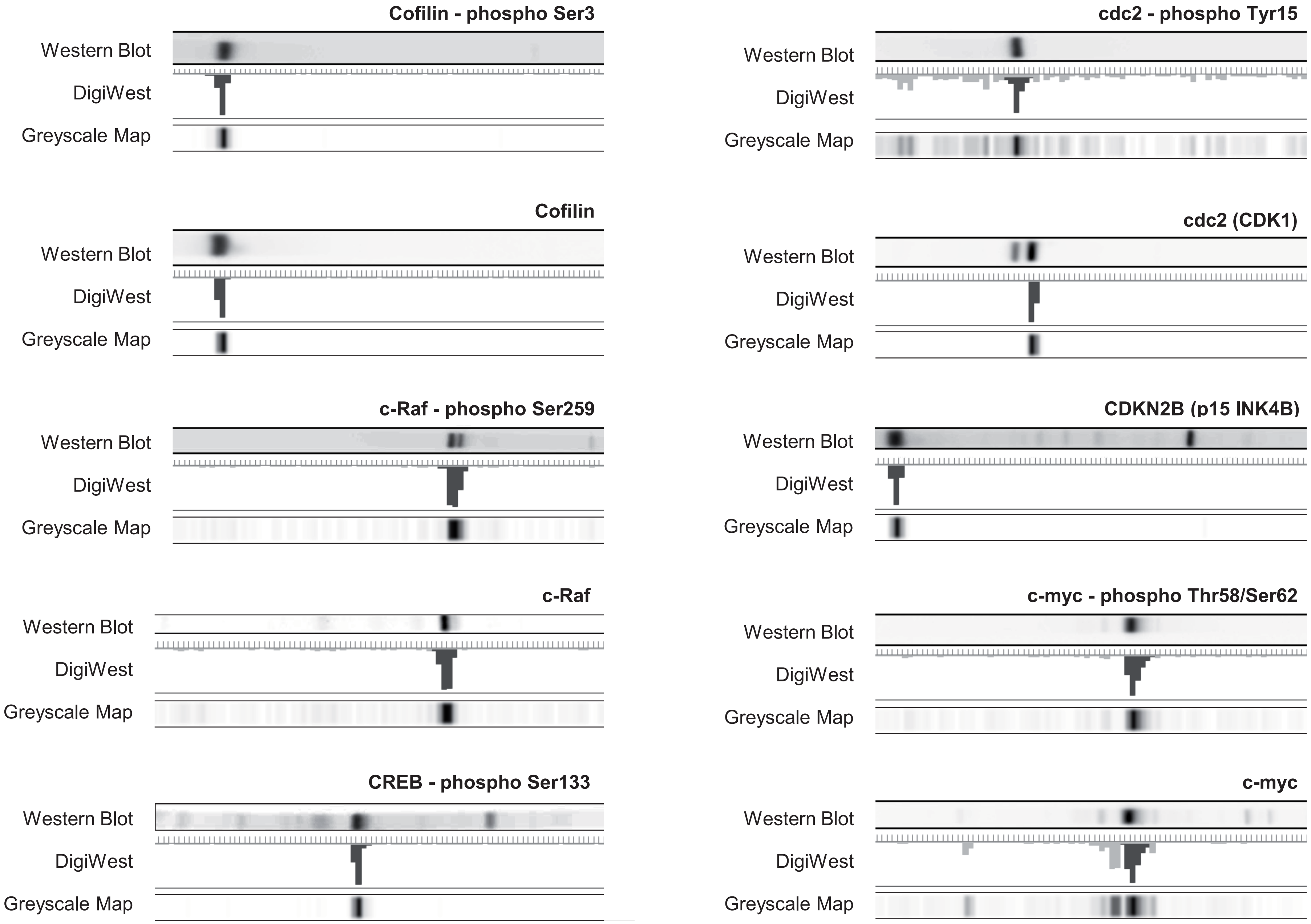

Cofilin - phospho Ser3
Western Blot
DigiWest
Greyscale Map
Cofilin
Western Blot
DigiWest
Greyscale Map
c-Raf - phospho Ser259
Western Blot
DigiWest
Greyscale Map
c-Raf
Western Blot
DigiWest
Greyscale Map
CREB - phospho Ser133
Western Blot
DigiWest
Greyscale Map
cdc2 - phospho Tyr15
Western Blot
DigiWest
Greyscale Map
cdc2 (CDK1)
Western Blot
DigiWest
Greyscale Map
CDKN2B (p15 INK4B)
Western Blot
DigiWest
Greyscale Map
c-myc - phospho Thr58/Ser62
Western Blot
DigiWest
Greyscale Map
c-myc
Western Blot
DigiWest
Greyscale Map

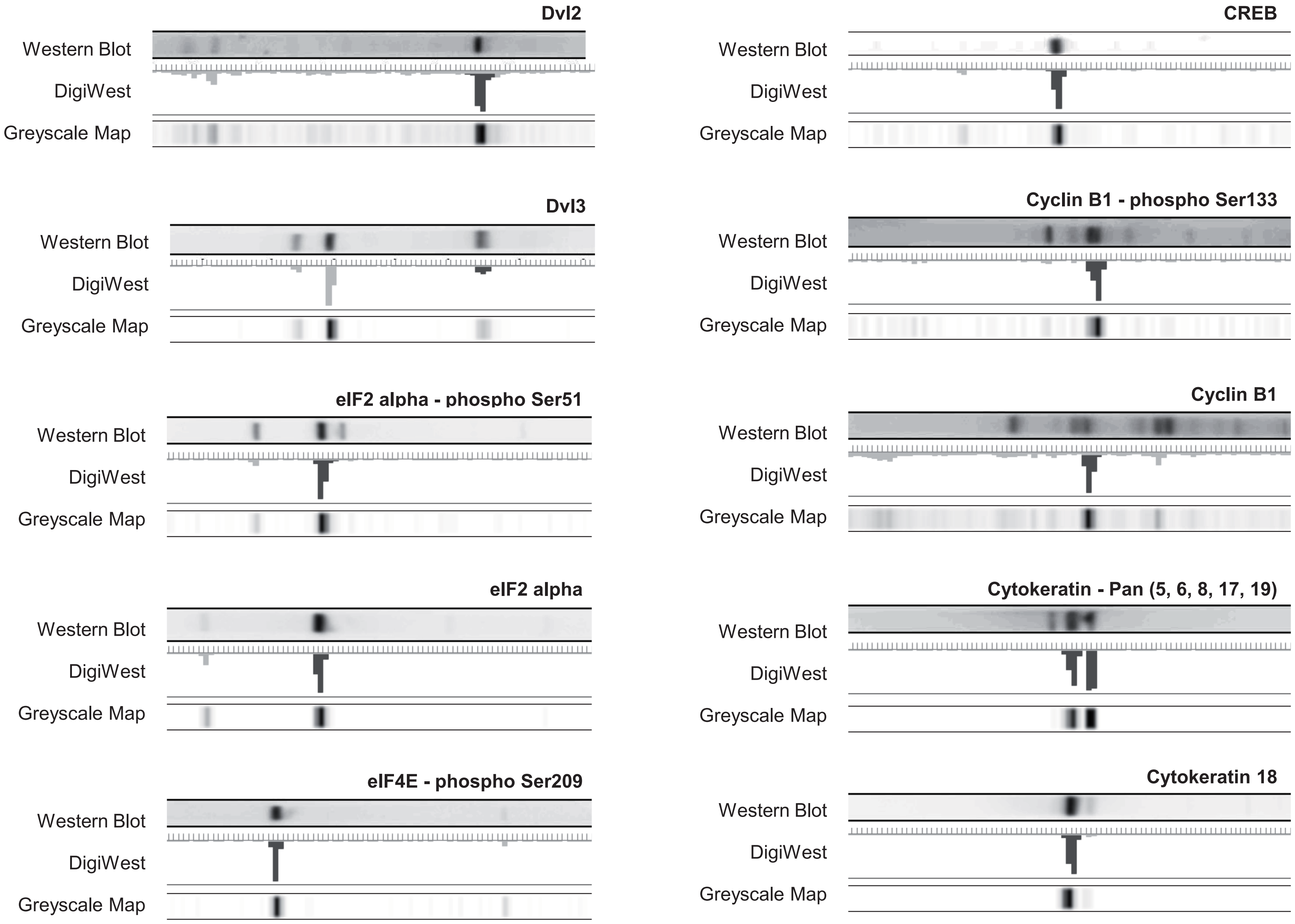
Dvl2
Western Blot
DigiWest
Greyscale Map
Dvl3
Western Blot
DigiWest
Greyscale Map
eIF2 alpha - phospho Ser51
Western Blot
DigiWest
Greyscale Map
eIF2 alpha
Western Blot
DigiWest
Greyscale Map
eIF4E - phospho Ser209
Western Blot
DigiWest
Greyscale Map
CREB
Western Blot
DigiWest
Greyscale Map
Cyclin B1 - phospho Ser133
Western Blot
DigiWest
Greyscale Map
Cyclin B1
Western Blot
DigiWest
Greyscale Map
Cytokeratin - Pan (5, 6, 8, 17, 19)
Western Blot
DigiWest
Greyscale Map
Cytokeratin 18
Western Blot
DigiWest
Greyscale Map

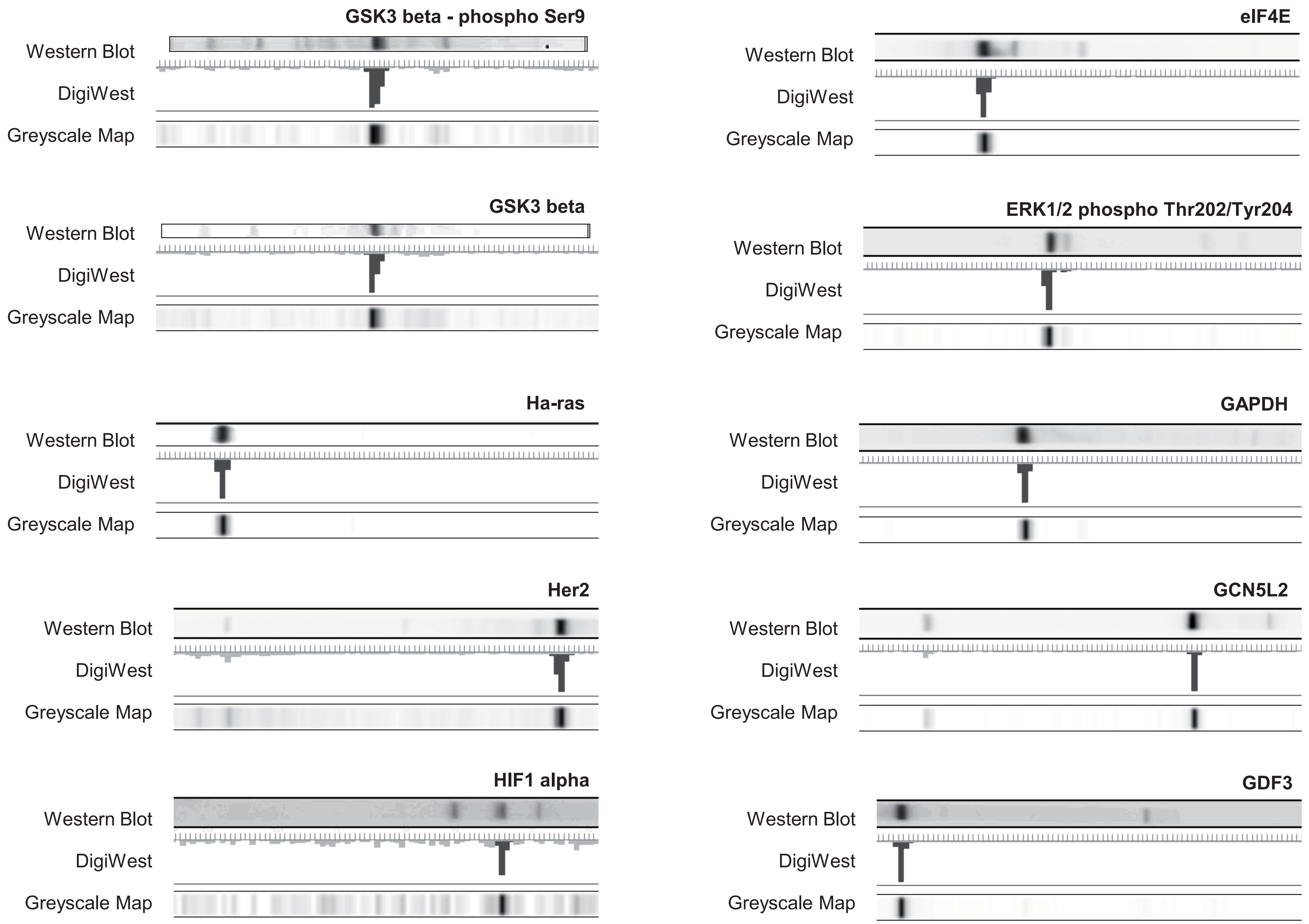
GSK3 beta - phospho Ser9
Western Blot
DigiWest
Greyscale Map
GSK3 beta
Western Blot
DigiWest
Greyscale Map
Ha-ras
Western Blot
DigiWest
Greyscale Map
Her2
Western Blot
DigiWest
Greyscale Map
HIF1 alpha
Western Blot
DigiWest
Greyscale Map
eIF4E
Western Blot
DigiWest
Greyscale Map
ERK1/2 phospho Thr202/Tyr204
Western Blot
DigiWest
Greyscale Map
GAPDH
Western Blot
DigiWest
Greyscale Map
GCN5L2
Western Blot
DigiWest
Greyscale Map
GDF3
Western Blot
DigiWest
Greyscale Map

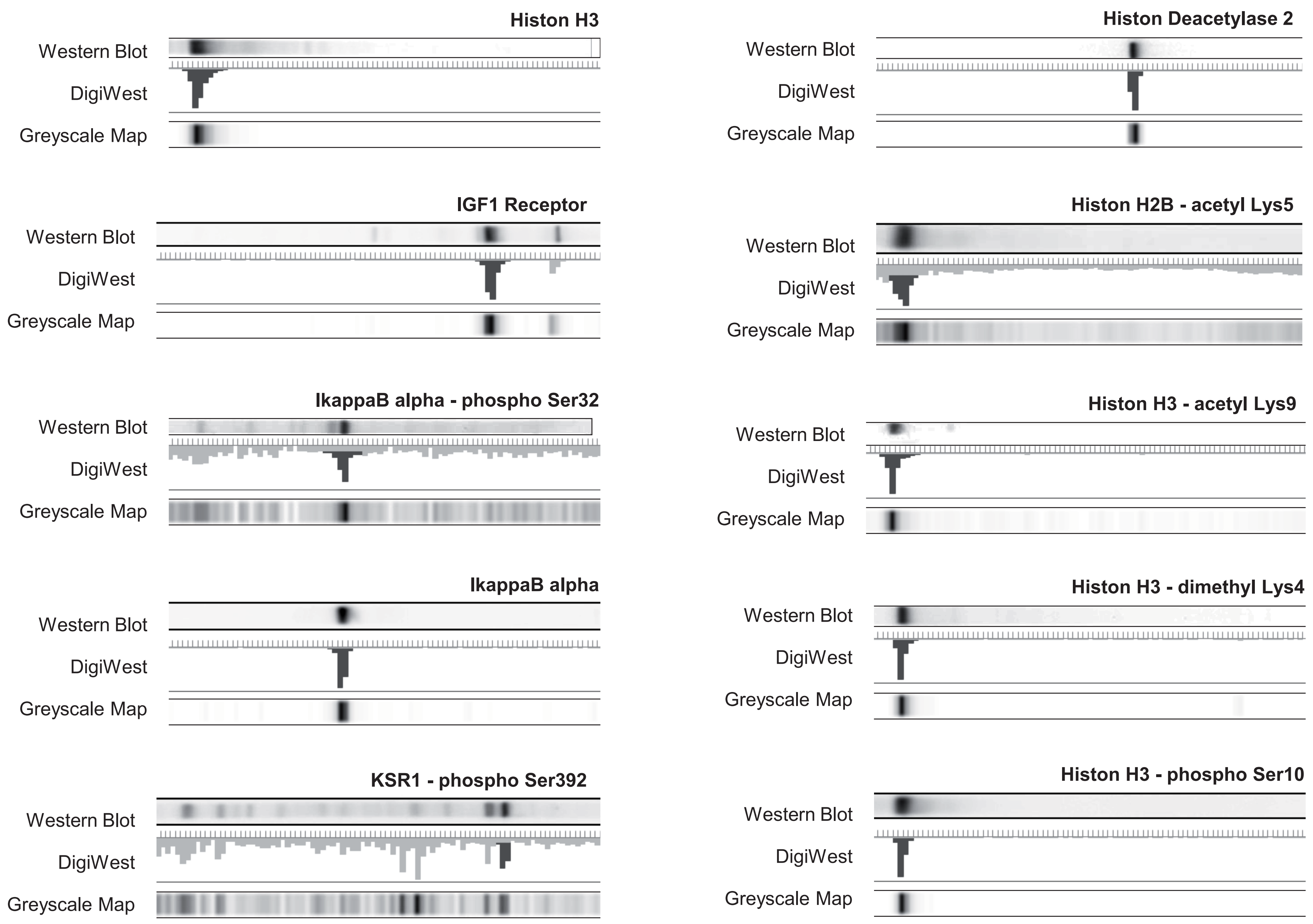
Histon H3
Western Blot
DigiWest
Greyscale Map
IGF1 Receptor
Western Blot
DigiWest
Greyscale Map
IkappaB alpha - phospho Ser32
Western Blot
DigiWest
Greyscale Map
IkappaB alpha
Western Blot
DigiWest
Greyscale Map
KSR1 - phospho Ser392
Western Blot
DigiWest
Greyscale Map
Histon Deacetylase 2
Western Blot
DigiWest
Greyscale Map
Histon H2B - acetyl Lys5
Western Blot
DigiWest
Greyscale Map
Histon H3 - acetyl Lys9
Western Blot
DigiWest
Greyscale Map
Histon H3 - dimethyl Lys4
Western Blot
DigiWest
Greyscale Map
Histon H3 - phospho Ser10
Western Blot
DigiWest
Greyscale Map

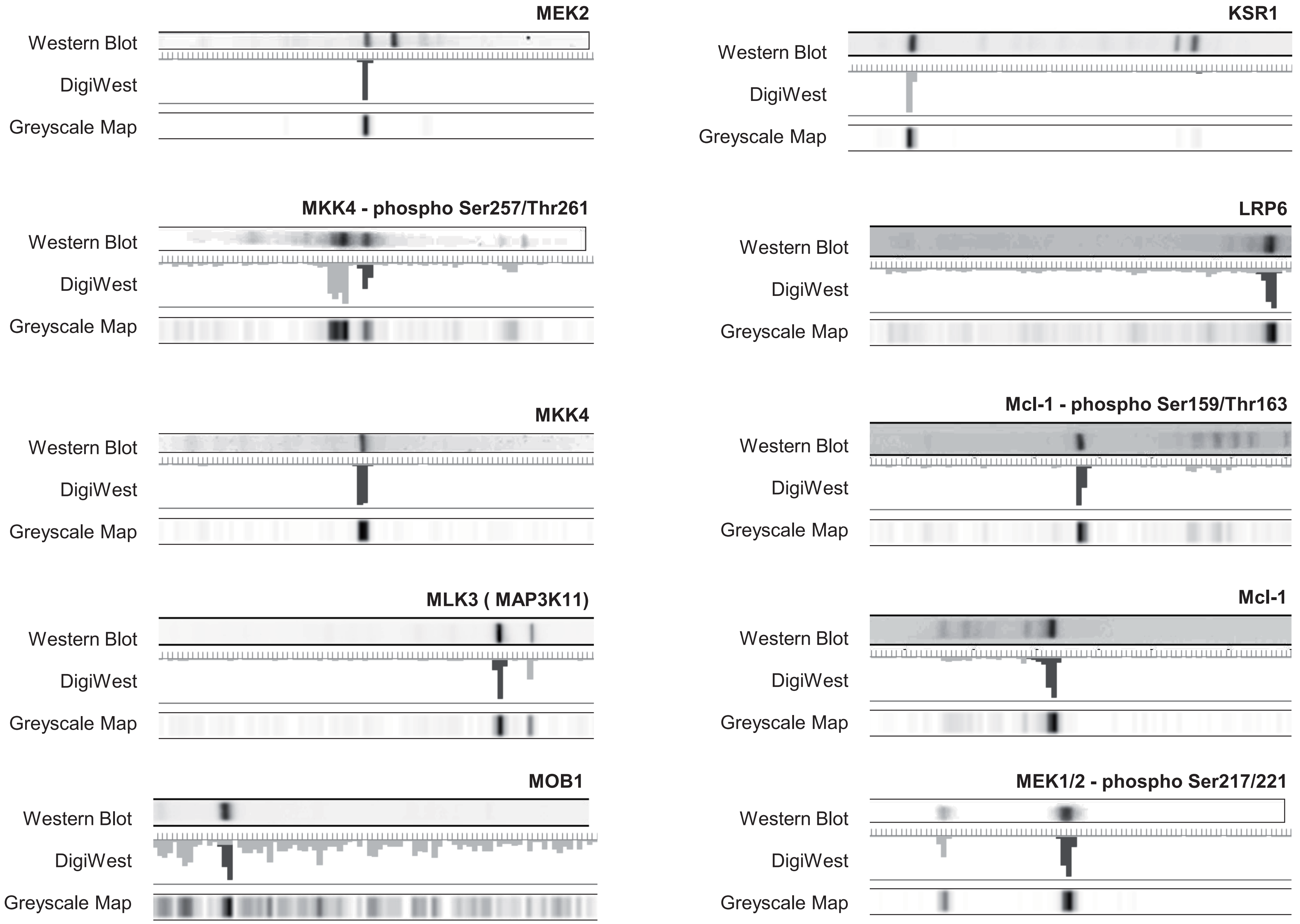

MEK2
Western Blot
DigiWest
Greyscale Map
KSR1
Western Blot
DigiWest
Greyscale Map
MKK4 - phospho Ser257/Thr261
Western Blot
DigiWest
Greyscale Map
LRP6
Western Blot
DigiWest
Greyscale Map
MKK4
Western Blot
DigiWest
Greyscale Map
Mcl-1 - phospho Ser159/Thr163
Western Blot
DigiWest
Greyscale Map
MLK3 (MAP3K11)
Western Blot
DigiWest
Greyscale Map
Mcl-1
Western Blot
DigiWest
Greyscale Map
MOB1
Western Blot
DigiWest
Greyscale Map
MEK1/2 - phospho Ser217/221
Western Blot
DigiWest
Greyscale Map

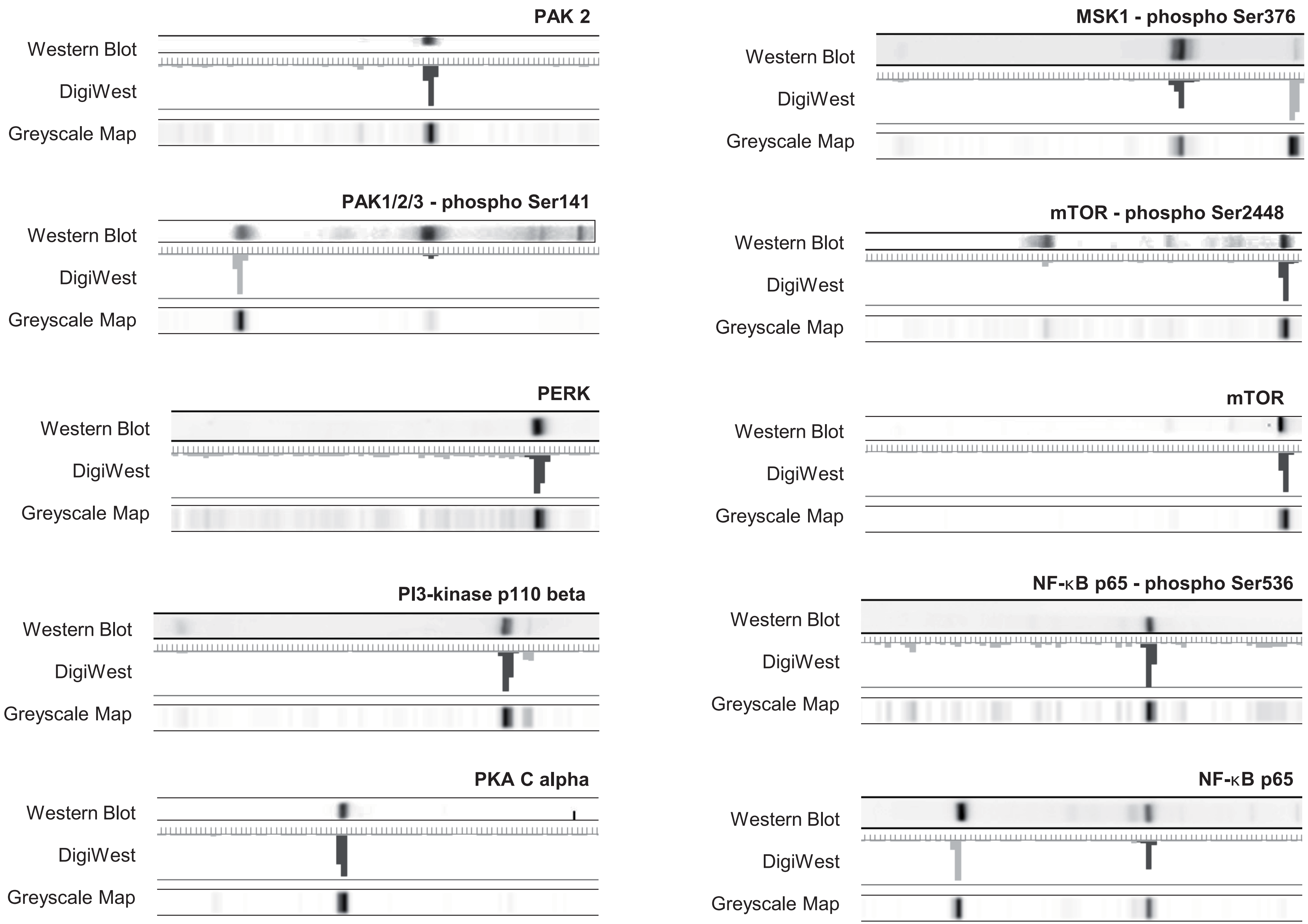
PAK 2
Western Blot
DigiWest
Greyscale Map
PAK1/2/3 - phospho Ser141
Western Blot
DigiWest
Greyscale Map
PERK
Western Blot
DigiWest
Greyscale Map
PI3-kinase p110 beta
Western Blot
DigiWest
Greyscale Map
PKA C alpha
Western Blot
DigiWest
Greyscale Map
MSK1 - phospho Ser376
Western Blot
DigiWest
Greyscale Map
mTOR - phospho Ser2448
Western Blot
DigiWest
Greyscale Map
mTOR
Western Blot
DigiWest
Greyscale Map
NF-κB p65 - phospho Ser536
Western Blot
DigiWest
Greyscale Map
NF-κB p65
Western Blot
DigiWest
Greyscale Map

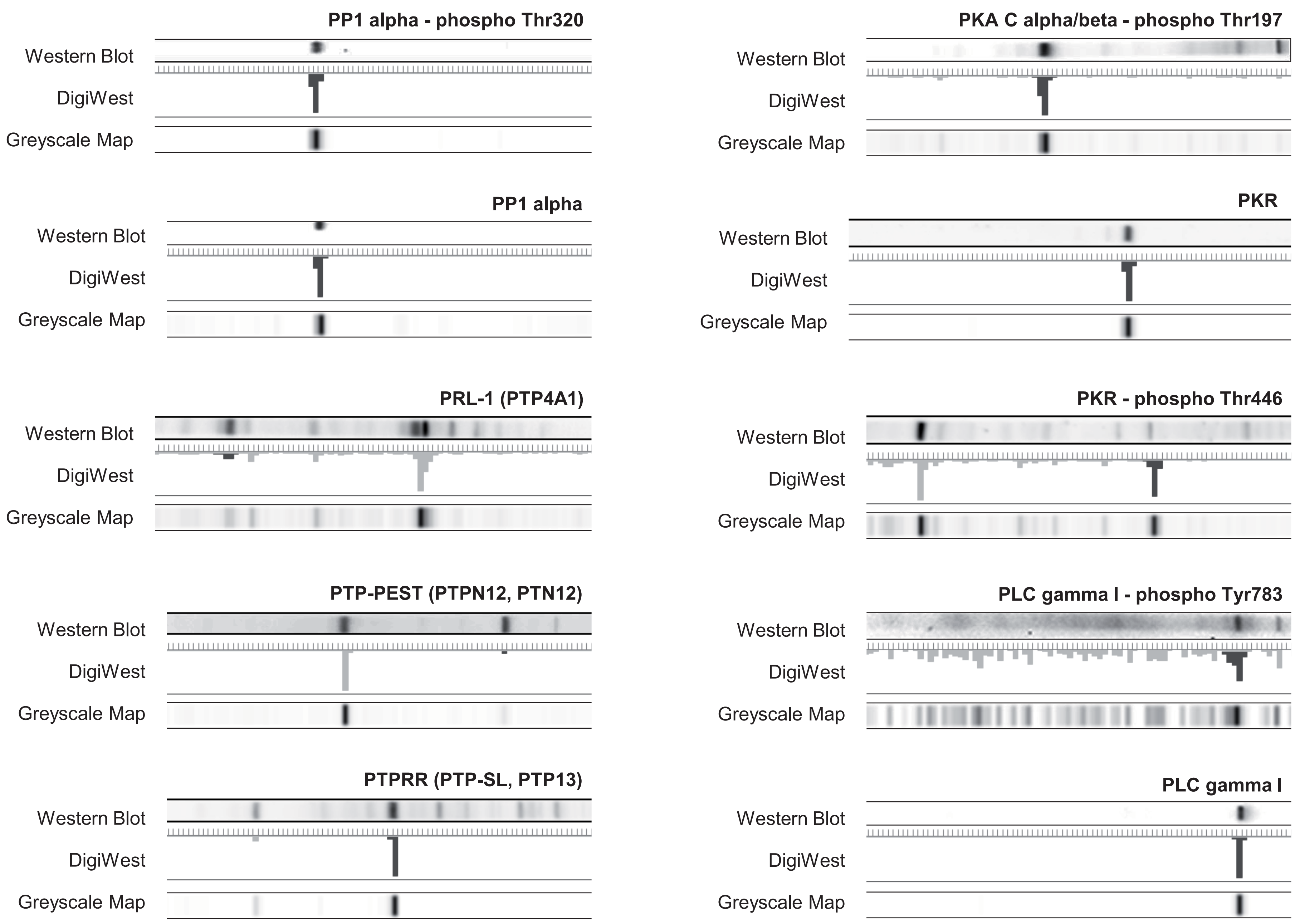
PP1 alpha - phospho Thr320
Western Blot
DigiWest
Greyscale Map
PP1 alpha
Western Blot
DigiWest
Greyscale Map
PRL-1 (PTP4A1)
Western Blot
DigiWest
Greyscale Map
PTP-PEST (PTPN12, PTN12)
Western Blot
DigiWest
Greyscale Map
PTPRR (PTP-SL, PTP13)
Western Blot
DigiWest
Greyscale Map
PKA C alpha/beta - phospho Thr197
Western Blot
DigiWest
Greyscale Map
PKR
Western Blot
DigiWest
Greyscale Map
PKR - phospho Thr446
Western Blot
DigiWest
Greyscale Map
PLC gamma I - phospho Tyr783
Western Blot
DigiWest
Greyscale Map
PLC gamma I
Western Blot
DigiWest
Greyscale Map

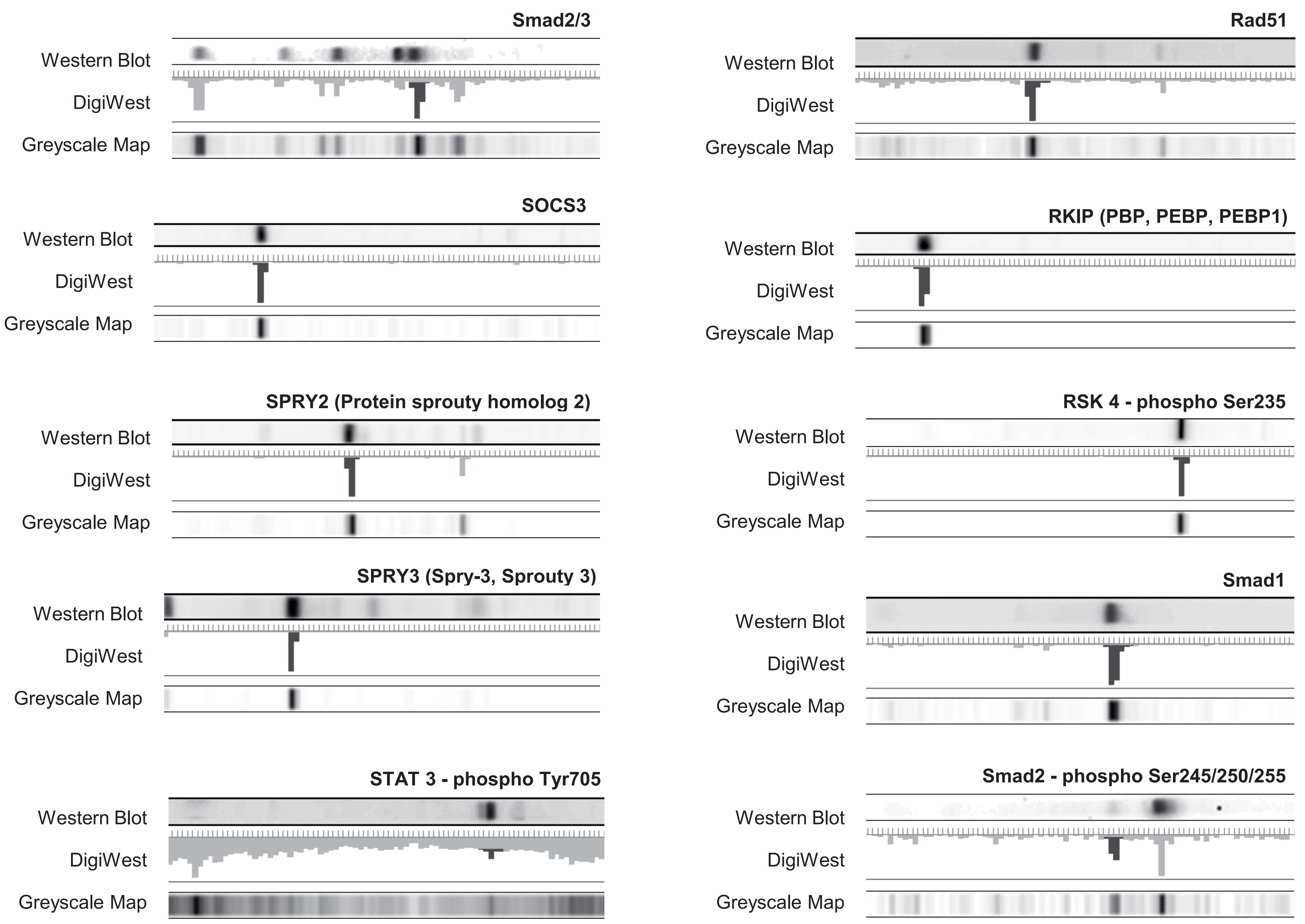
Smad2/3
Western Blot
DigiWest
Greyscale Map
SOCS3
Western Blot
DigiWest
Greyscale Map
SPRY2 (Protein sprouty homolog 2)
Western Blot
DigiWest
Greyscale Map
SPRY3 (Spry-3, Sprouty 3)
Western Blot
DigiWest
Greyscale Map
STAT 3 - phospho Tyr705
Western Blot
DigiWest
Greyscale Map
Rad51
Western Blot
DigiWest
Greyscale Map
RKIP (PBP, PEBP, PEBP1)
Western Blot
DigiWest
Greyscale Map
RSK 4 - phospho Ser235
Western Blot
DigiWest
Greyscale Map
Smad1
Western Blot
DigiWest
Greyscale Map
Smad2 - phospho Ser245/250/255
Western Blot
DigiWest
Greyscale Map

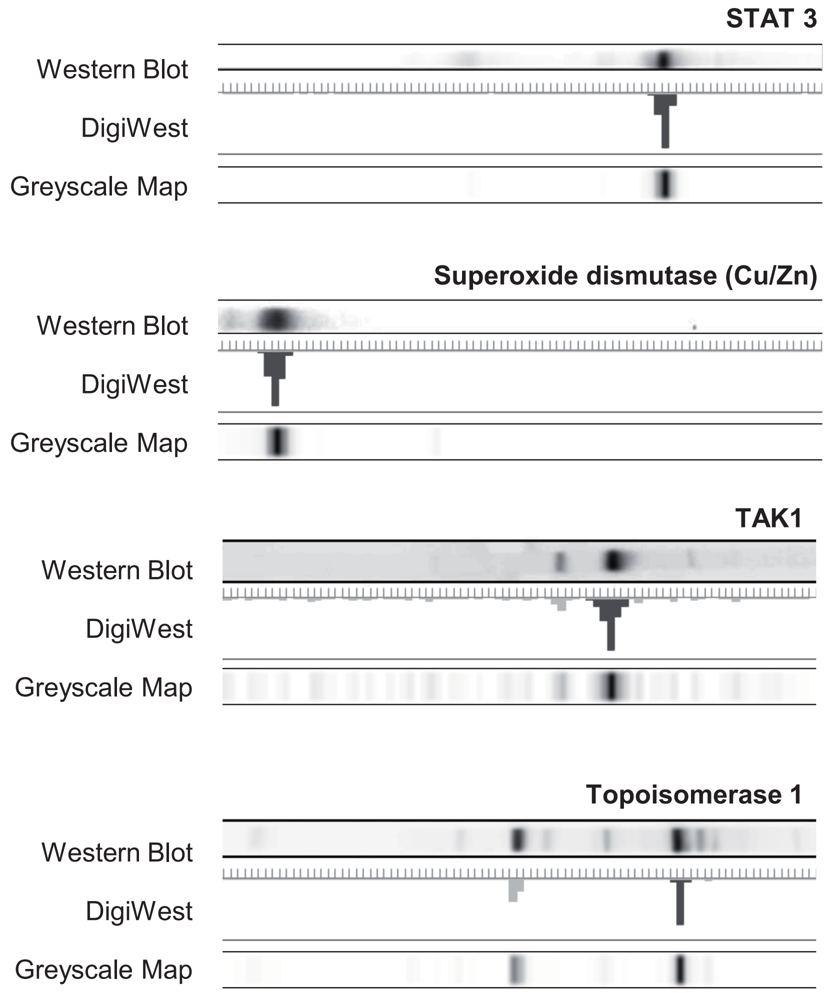
STAT 3
Western Blot
DigiWest
Greyscale Map
Superoxide dismutase (Cu/Zn)
Western Blot
DigiWest
Greyscale Map
TAK1
Western Blot
DigiWest
Greyscale Map
Topoisomerase 1
Western Blot
DigiWest
Greyscale Map

Supplementary figure 2: Effect of protein biotinylation on antibody binding during Western-blotting.

Western-blots were performed under standard conditions and 20 µg HepG2 lysate were loaded per lane. Half of the Western-blot was processed under standard conditions, whereas the other half was subjected to the biotinylation step as performed in the DigiWest workflow. The Western-blots were cut into blocks each containing 2 lanes and a molecular weight marker and antibody incubations were performed each using one block of a blot with and without biotinylation. Antibodies detecting proteins of different molecular weight and antibodies detecting phosphorylated proteins were used. To verify protein biotinylation, one block with and without biotinylation was incubated with IRDye800 labelled streptavidin.
No differences in signal intensities for the 12 tested antibodies were detected, indicating that the biotinylation step that is performed during the DigiWest procedure does not influence antigenicity of the separated proteins and antibody recognition is not influenced.

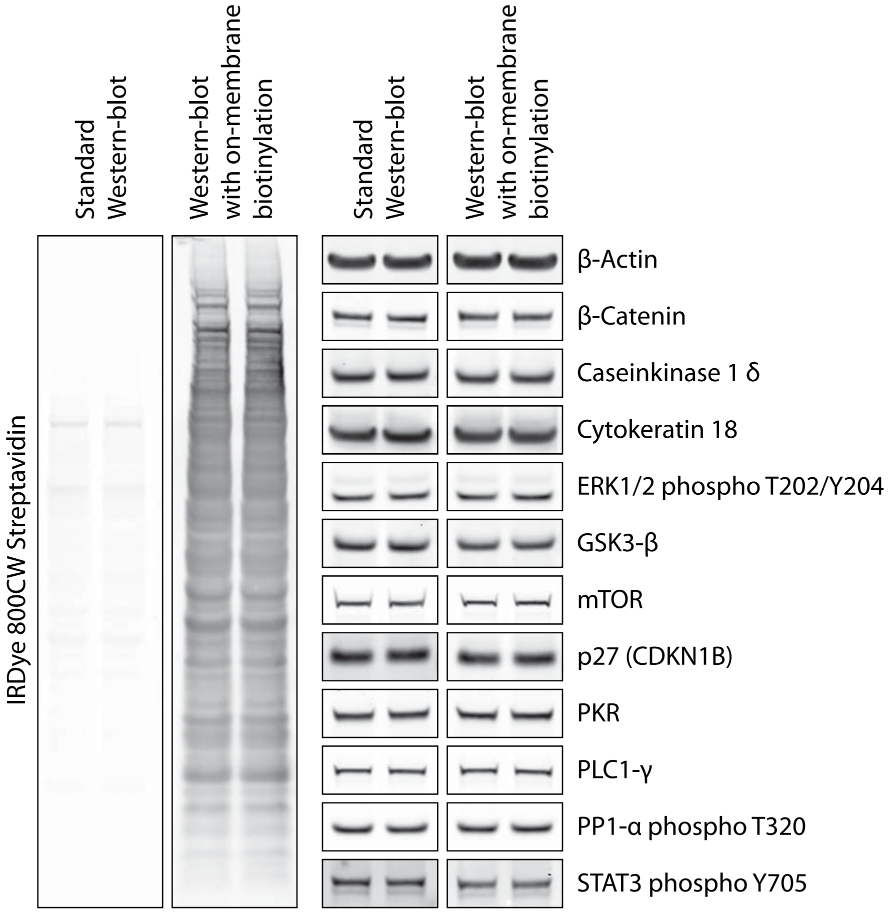

Supplementary figure 3: Comparison of DigiWest and Western blot – Correlation analysis.

Correlation analysis between DigiWest peak intensities and Western blot band intensities was performed on expression data generated by the described DigiWest procedure and by a standarised Western blot protocol. Western blot band intensities were quantified using a near infrared fluorescence scanner as read-out system (see methods section). Proteins from mouse liver lysate (10 µg) were separated for DigiWest and for standard Western blotting, 30 antibodies (see supplementary figure 1) were and the obtained numerical data were analysed. 2 antibodies gave no band in Western blotting whereas for 1 antibody no signal in DigiWest and Western blot was obtained. The signals generated for the remaining 27 antibodies were used for a correlation analysis and a Pearson correlation coefficient r of 0.86 was determined.

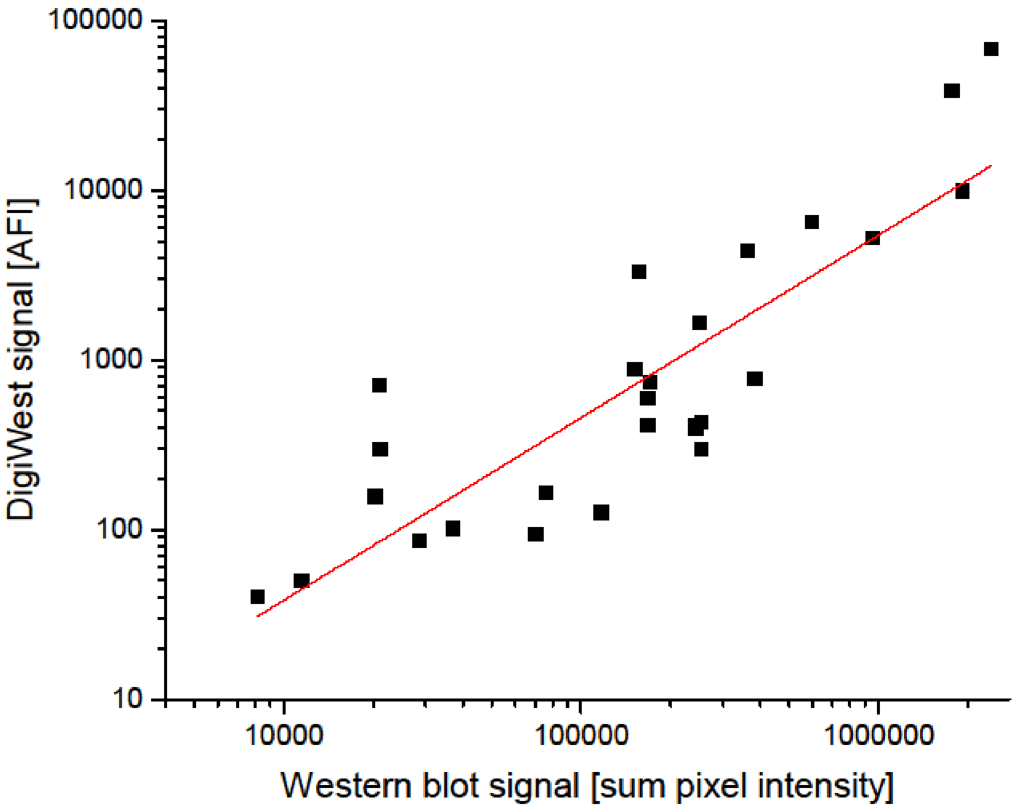

Supplementary figure 4: Binding capacity of Neutravidin coated beads.

To investigate the biotin binding capacity of the Neutravidin coated Luminex beads a Digi-West experiment was performed with a 1:2 dilution series of HepG2 lysate. 48 molecular weight fractions were generated from each Western blot lane covering the molecular weight range from 30 to 110 kDa containing most of the highly abundant proteins. After protein loading the DigiWest beads were incubated with a biotinylated peptide (c-Myc, Biotin-Doa-Doa-EQKLISEEDL) to occupy the remaining free biotin binding sites. The resulting bead mixes were either incubated with PE-labelled streptavidin or with an antibody detecting the peptide (c-Myc clone 9E10) and a PE-labelled secondary antibody. The signal obtained with PE-labelled streptavidin was used as a measure for bead-bound biotinylated proteins where-as the signal obtained with the c-Myc antibody was used as a measure for free biotin binding sites.

a) Signal obtained with PE-labelled streptavidin (red) normalized to the highest value and signal obtained with the c-Myc antibody (blue) normalized to the highest value (negative values). When 40 µg HepG2 lysate are loaded saturation occurs based on the biotin-binding capacity.

b) Integrated signals normalized to the highest value.

Under the established DigiWest condition (amount of loaded protein 2.5 to 20 µg) the binding capacity of the Neutravidin coated Luminex beads is in excess of biotinylated protein and thereby quantitative measurements are possible.

a)

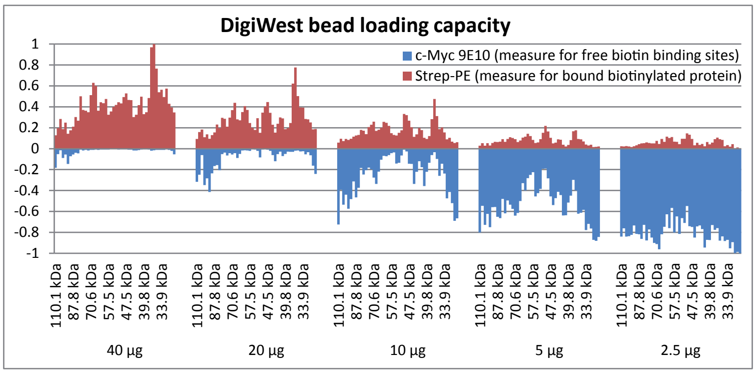

b)

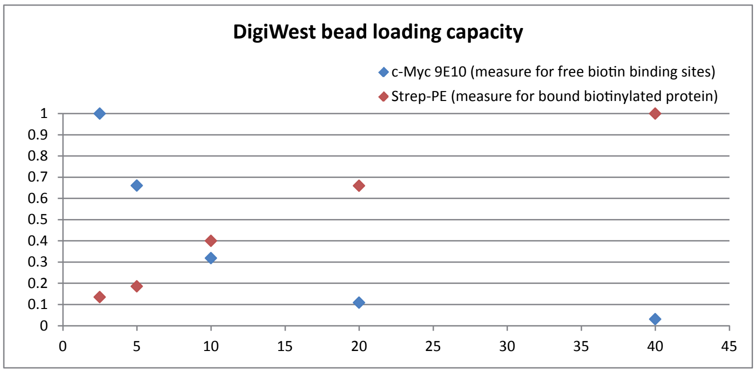

Supplementary figure 5: Protein binding capacity: Effect of increasing amounts of competing protein on signal intensity.

Beads obtained from the spike-in experiment (supplementary figure 2) were probed with an antibody recognizing PKR, a protein running exactly at the same molecular weight as GST-ERK2 (69 kDa) and signal was generated using standard conditions. Also shown is the signal generated with the ERK1/2 specific antibody for the samples spiked with GST-ERK2 (replicate 1 in supplementary figure 2).

a) Representation of obtained signal for the antibody directed against ERK1/2 as bar diagram; intrinsic ERK1 is highlighted in black, intrinsic ERK2 is highlighted in dark gray, spiked GST-ERK2 in medium gray (data also shown in supplementary figure 2) and data obtained with the antibody directed against PKR is shown in black.

b) Western-blot mimics (gray-scale maps generated from the Luminex data) representing the same data as in a).

The standard deviation of all integrals of the obtained PKR signals showed a CV of 8.8%; no competing effect was observed by increasing the amount of spiked protein. This experiment implies that quantitative measurements are possible since even a high amount of competing protein does not influence signal intensity of the protein of interest.

Supplementary figure 5

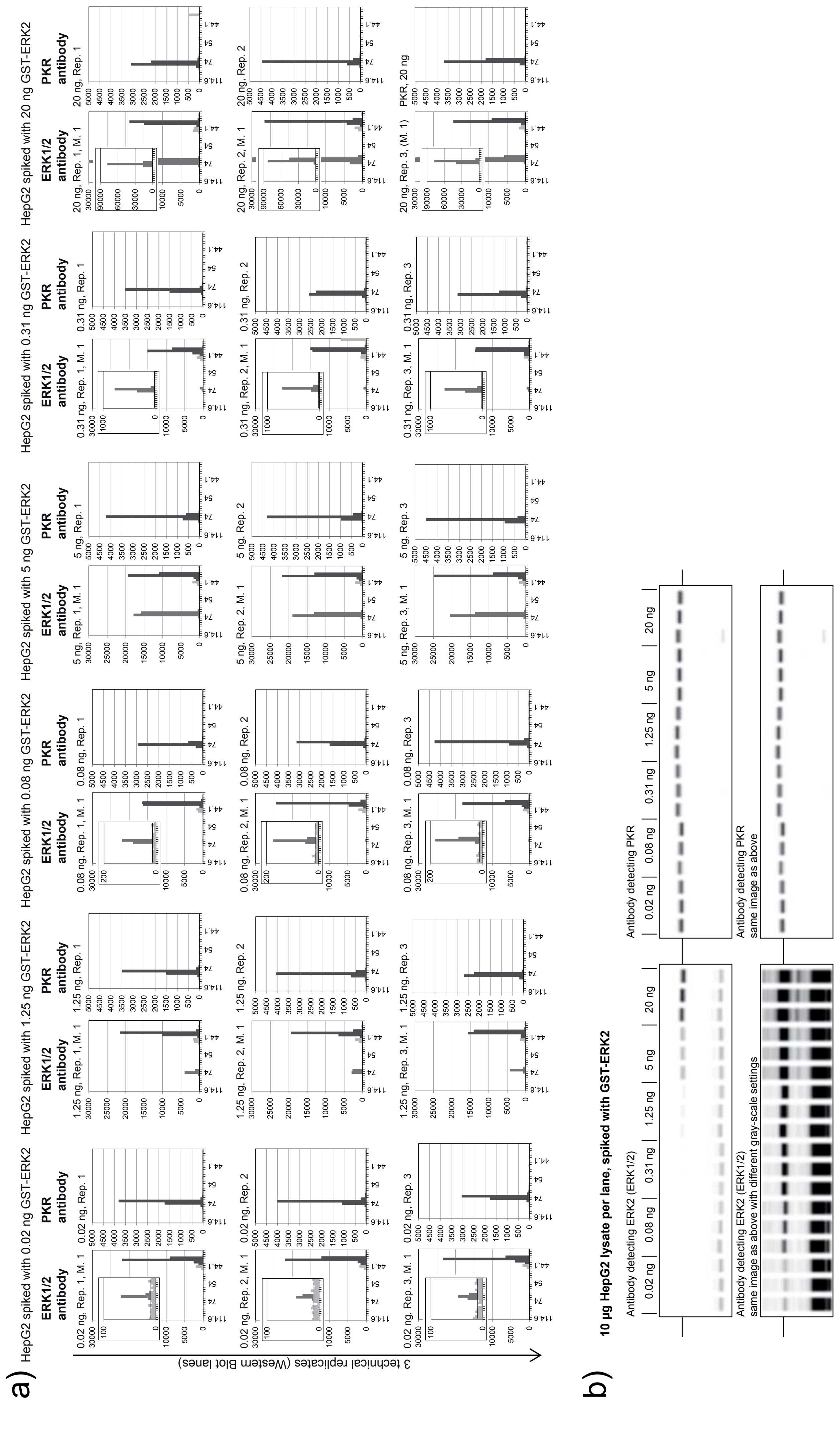

Supplementary figure 6: Spike-in experiment to determine sensitivity, linearity and reproducibility of DigiWest.

HepG2 lysate (10 µg per lane) was spiked with different amounts of GST-ERK2 (20 pg, 78 pg, 312 pg, 1.25 ng, 5 ng, 20 ng) and triplicate samples were generated. The 18 spiked samples were separated on three SDS-PAGE gels and DigiWest was performed for proteins with an apparent molecular weight between 40 kDa and 110 kDa. Bead-pools sufficient for 100 antibody incubations were prepared and aliquots were used for signal generation using an ERK1/2 specific antibody; measurements were repeated four times. The diagrams show intrinsic ERK2 highlighted in dark grey, GST-ERK2 in medium grey and ERK1 and remaining signal in light grey. Repeat measurements are shown from left to right whereas the technical replicates are shown from top to bottom.

The accompanying table shows the integrals of the identified peaks (GST-ERK2, intrinsic ERK2) obtained from DigiWest; the lower part of the table shows signal intensity obtained from quantification of the classical Western-Blot image.

Supplementary figure 6

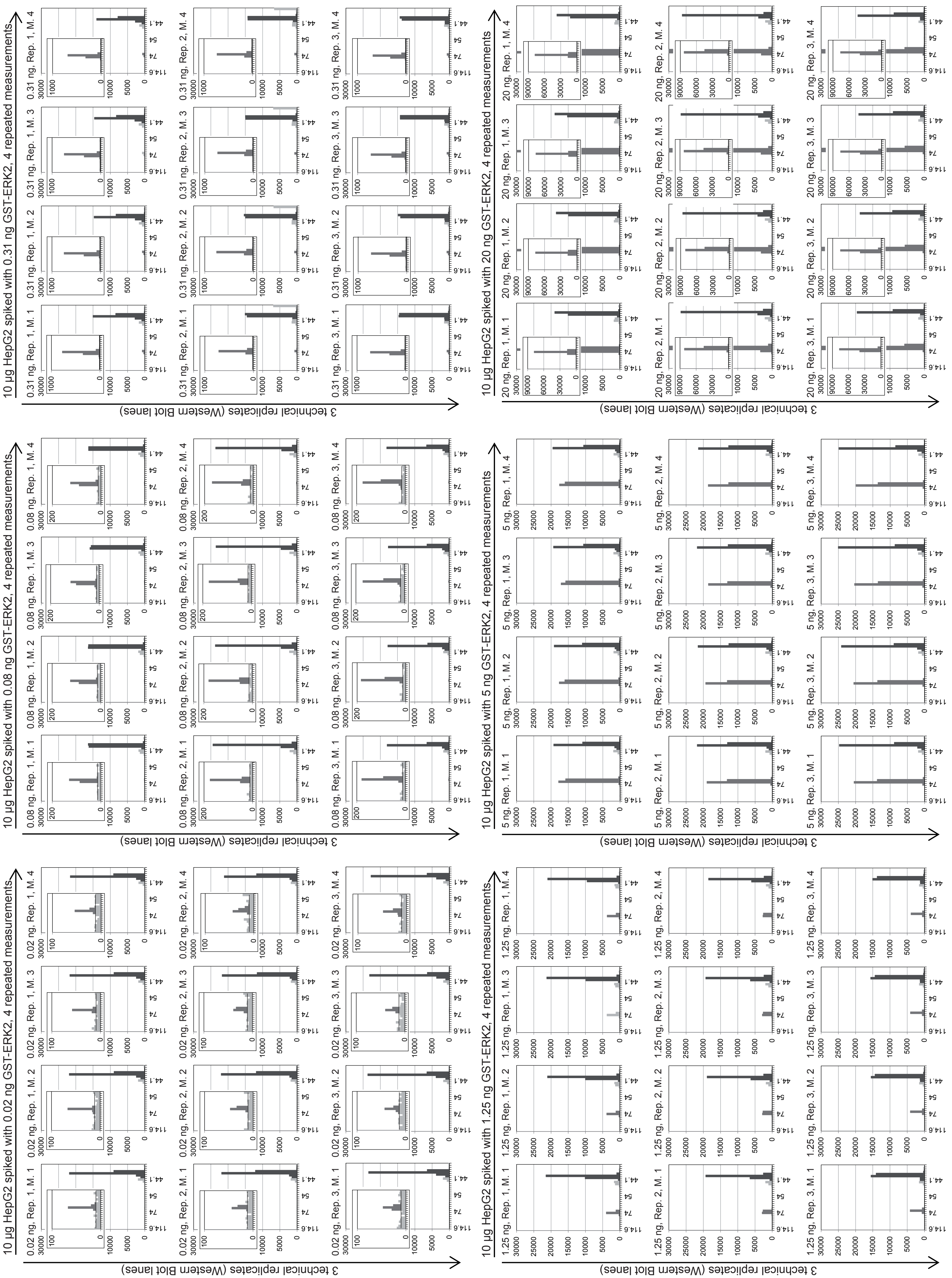

DigiWest

GST-ERK2 quantification

(Spike-in per 10 µg HepG2 Lysate, relative signal)

		Replicate 1	Replicate 2	Replicate 3	Mean	CV (%)
20 ng	Meas. 1	111610	132187	123061		
	Meas. 2	110545	131069	121013	121546	7.58
	Meas. 3	109539	133063	121319		
	Meas. 4	110796	132258	122093		
5 ng	Meas. 1	34446	32902	35241		
	Meas. 2	34452	33242	35176	33923	3.15
	Meas. 3	33635	32368	34849		
	Meas. 4	34339	32063	34365		
1.25 ng	Meas. 1	5458	5860	5568		
	Meas. 2	5339	5771	5471	5469	4.37
	Meas. 3	5212	5727	5450		
	Meas. 4	5095	5503	5178		
0.31 ng	Meas. 1	1208	993	1118		
	Meas. 2	1159	1051	1159	1097	6.02
	Meas. 3	1149	1046	1117		
	Meas. 4	1074	1006	1086		
0.08 ng	Meas. 1	217	241	270		
	Meas. 2	215	275	260	249	10.91
	Meas. 3	228	252	264		
	Meas. 4	215	251	302		
0.02 ng	Meas. 1	85	60	73		
	Meas. 2	78	75	77	73	9.38
	Meas. 3	67	64	74		
	Meas. 4	73	71	79		

Intrinsic ERK2

(in 10 µg HepG2 lysate, relative signal)

		Replicate 1	Replicate 2	Replicate 3
20 ng	Meas. 1	34953	33795	29330
	Meas. 2	34518	33194	28654
	Meas. 3	35208	33441	28729
	Meas. 4	33745	33003	30158
5 ng	Meas. 1	32769	37621	35915
	Meas. 2	32763	36971	35567
	Meas. 3	32522	37395	36033
	Meas. 4	32608	36794	35699
1.25 ng	Meas. 1	33006	29025	32337
	Meas. 2	32512	28286	32558
	Meas. 3	32573	28731	32674
	Meas. 4	31866	27581	31418
0.31 ng	Meas. 1	27349	31321	32156
	Meas. 2	26813	32022	31902
	Meas. 3	26737	31581	31398
	Meas. 4	25480	30164	30841
0.08 ng	Meas. 1	33943	31263	27490
	Meas. 2	34069	30510	27039
	Meas. 3	32726	30317	27053
	Meas. 4	33933	30173	27490
0.02 ng	Meas. 1	34688	37989	34494
	Meas. 2	34682	37824	34829
	Meas. 3	34465	37132	34060
	Meas. 4	34419	36704	33692

Mean	CV (%)
32399	9.57

Western-Blot on Licor Odyssey

GST-ERK2 quantification

(Spike-in per 10 µg HepG2 Lysate, relative signal)

	Replicate 1	Replicate 2	Replicate 3	Mean	CV%
20 ng	318473100	329781002		324127051	NA
5 ng	135073639	134463994		134768817	NA
1.25 ng	36316470	36196382		36256426	NA
0.31 ng	8911814	9002800	9625102	9179905	4.23
0.08 ng	2586263	2532097	2636531	2584964	2.02
0.02 ng	1108895	1177614	924245	1070252	12.24

Intrinsic ERK2 (in 10 µg HepG2 lysate)

(in 10 µg HepG2 lysate, relative signal)

	Replicate 1	Replicate 2	Replicate 3
20 ng	106277100	107386932	
5 ng	108356255	109254315	
1.25 ng	108724000	109124584	
0.31 ng	115500031	121316792	124221951
0.08 ng	125092496	122582478	119270453
0.02 ng	123804788	126355252	123822790

Mean	CV%
116739348	6.58

Supplementary figure 7: Generation of Lapatinib resistant H292 cells, IC50.

To evoke resistance, H292 cells were cultured in the presence of increasing concentrations of Lapatinib for approximately 7 months. A cell viability assay shows that H292 parental cells are responsive to Lapatinib in the low nanomolar range (IC50 ~ 10nM), whereas resistant cells tolerate 80 fold higher doses (IC50 ~ 800nM).

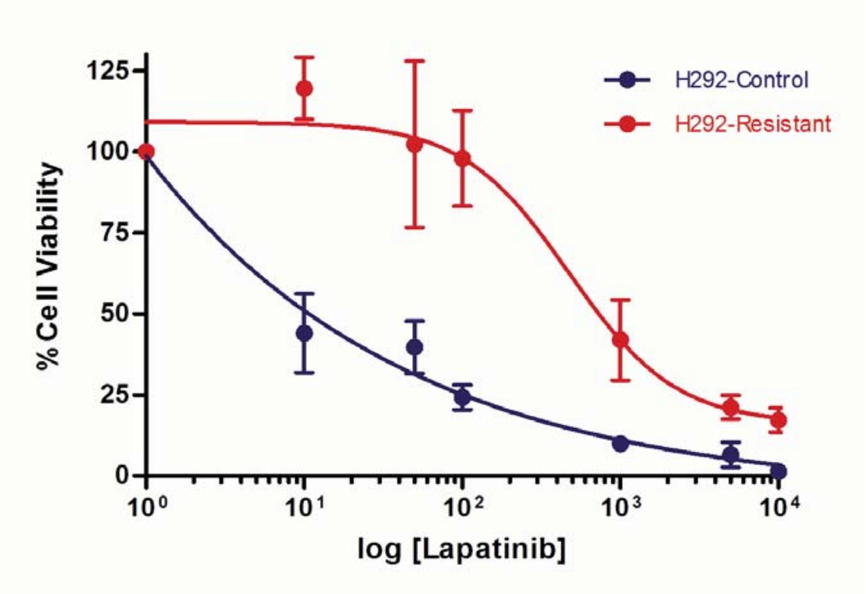

IC_{50} Control ca. 10nM

IC_{50} Resistant ca. 800nM

Supplementary figure 8: H292 Lapatinib resistance: Western-Blot mimics of significant kinases.

Western-blot mimics generated from the data obtained on the Kinobead pulldowns from the Lapatinib resistant and the parental H292 cell lysates. Represented are all kinases which are shown in figure 4b and 4c which showed significant changes ($p < 0.01$, log2 > 1).

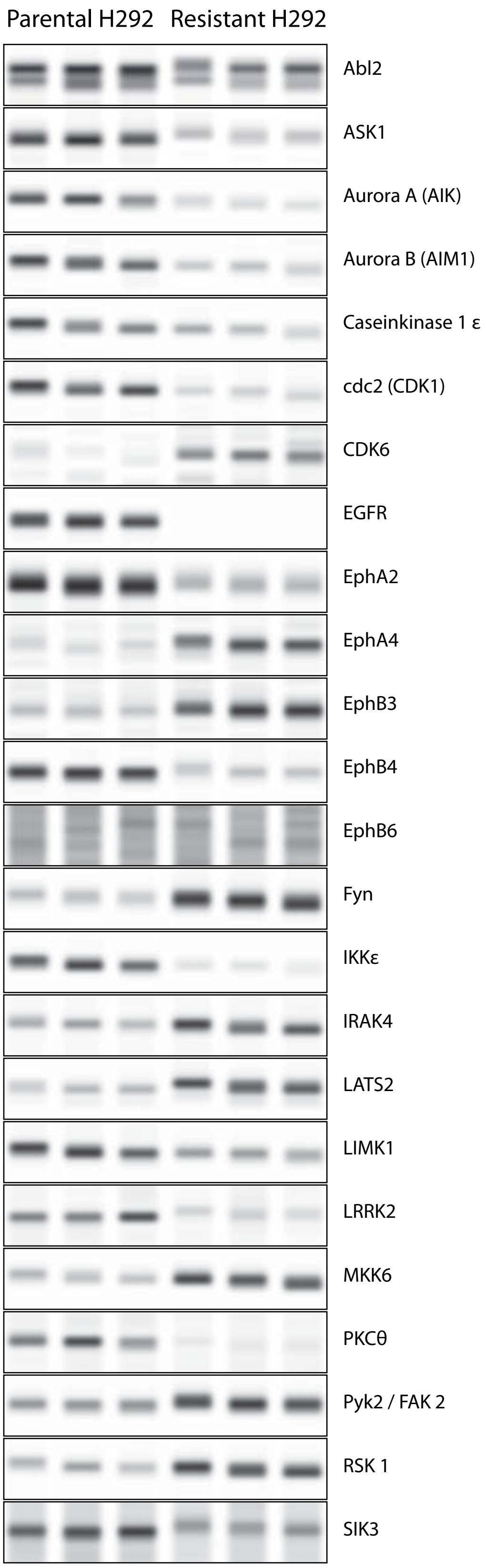

Supplementary figure 9: H292 Lapatinib resistance: Canonical pathway and regulator analysis.

Shown are additional details from the Ingenuity pathway analysis for changes in the Lapatinib resistant H292 cell line compared to the parental cell line. Details on the analysis are provided in the methods section. Top canonical pathways associated with the uploaded dataset were extracted. Next, an upstream regulator analysis was performed in order to identify the cascade of upstream transcriptional regulators that are able to explain the observed gene expression changes in our dataset. Given are the top 5 canonical pathways and the top 5 regulators from the analysis.

Regulator Analysis

Top Canonical Pathways

Name	p-value	Overlap	
HER-2 Signaling in Breast Cancer	1.48E-12	10.5 %	8/76
Apoptosis Signaling	4.96E-12	9.1 %	8/88
Molecular Mechanisms of Cancer	4.69E-11	3.1 %	11/359
Cell Cycle: G2/M DNA Damage Checkpoint Regulation	4.49E-10	12.2 %	6/49
Cyclins and Cell Cycle Regulation	7.33E-09	7.8 %	6/77

Top Upstream Regulators

Upstream Regulator	p-value of overlap
TP53	2.05E-15
FOXM1	6.89E-11
NOTCH1	3.55E-07
BRCA1	1.87E-06
TOP3A	2.99E-06
